# Dynamics of gene silencing during X inactivation using allele-specific RNA-seq

**DOI:** 10.1186/s13059-015-0698-x

**Published:** 2015-08-03

**Authors:** Hendrik Marks, Hindrik H. D. Kerstens, Tahsin Stefan Barakat, Erik Splinter, René A. M. Dirks, Guido van Mierlo, Onkar Joshi, Shuang-Yin Wang, Tomas Babak, Cornelis A. Albers, Tüzer Kalkan, Austin Smith, Alice Jouneau, Wouter de Laat, Joost Gribnau, Hendrik G. Stunnenberg

**Affiliations:** Radboud University, Faculty of Science, Department of Molecular Biology, Radboud Institute for Molecular Life Sciences (RIMLS), 6500HB Nijmegen, The Netherlands; Radboud University, Faculty of Science, Department of Molecular Developmental Biology, Radboud Institute for Molecular Life Sciences (RIMLS), 6500HB Nijmegen, The Netherlands; Department of Reproduction and Development, Erasmus MC, University Medical Center, Rotterdam, The Netherlands; Hubrecht Institute, University Medical Center Utrecht, Uppsalalaan 8, 3584CT Utrecht, The Netherlands; Biology Department, Queen’s University, Kingston, ON Canada; Wellcome Trust-Medical Research Council Stem Cell Institute, University of Cambridge, Tennis Court Road, Cambridge, CB2 1QR UK; INRA, UMR1198 Biologie du Développement et Reproduction, F-78350 Jouy-en-Josas, France

## Abstract

**Background:**

During early embryonic development, one of the two X chromosomes in mammalian female cells is inactivated to compensate for a potential imbalance in transcript levels with male cells, which contain a single X chromosome. Here, we use mouse female embryonic stem cells (ESCs) with non-random X chromosome inactivation (XCI) and polymorphic X chromosomes to study the dynamics of gene silencing over the inactive X chromosome by high-resolution allele-specific RNA-seq.

**Results:**

Induction of XCI by differentiation of female ESCs shows that genes proximal to the X-inactivation center are silenced earlier than distal genes, while lowly expressed genes show faster XCI dynamics than highly expressed genes. The active X chromosome shows a minor but significant increase in gene activity during differentiation, resulting in complete dosage compensation in differentiated cell types. Genes escaping XCI show little or no silencing during early propagation of XCI. Allele-specific RNA-seq of neural progenitor cells generated from the female ESCs identifies three regions distal to the X-inactivation center that escape XCI. These regions, which stably escape during propagation and maintenance of XCI, coincide with topologically associating domains (TADs) as present in the female ESCs. Also, the previously characterized gene clusters escaping XCI in human fibroblasts correlate with TADs.

**Conclusions:**

The gene silencing observed during XCI provides further insight in the establishment of the repressive complex formed by the inactive X chromosome. The association of escape regions with TADs, in mouse and human, suggests that TADs are the primary targets during propagation of XCI over the X chromosome.

**Electronic supplementary material:**

The online version of this article (doi:10.1186/s13059-015-0698-x) contains supplementary material, which is available to authorized users.

## Background

Gene dosage of X-chromosomal genes in mammals is equalized between sexes by inactivation of one of the two X chromosomes in female cells [1]. During early embryonic development of mice, two waves of X chromosome inactivation (XCI) occur. At the two- to four-cell embryonic stage [embryonic day (E)1.5] the paternally derived X chromosome is inactivated, referred to as imprinted XCI. At the early blastocyst stage (E4.5) the X chromosome is reactivated, after which random XCI takes place: during a stochastic process either the maternally or paternally derived X chromosome is silenced (see Heard and Disteche [2], Barakat and Gribnau [3] and Jeon et al. [4] for comprehensive reviews). This second wave of random XCI can be recapitulated by in vitro differentiation of female mouse embryonic stem cells (ESCs), providing a powerful model system for studying XCI.

Random XCI is initiated through a regulatory interplay between two overlapping non-coding RNAs, Tsix and Xist. These genes are both positioned in the center of the X chromosome within the so-called X-inactivation center (XIC) [5]. Random XCI starts with the activation of Xist on the future inactivate X chromosome (Xi) and silencing of its negative regulator Tsix [6]. *Xist* subsequently accumulates over the future Xi in cis to induce silencing as further outlined below [7–9]. The X-encoded RNF12 (RLIM) is an important dose-dependent trans-acting XCI-activator at the onset of XCI [10–12]. Rnf12 is located in close proximity upstream of Xist and encodes a ubiquitin ligase, with REX1 as one of its main targets [13]. In undifferentiated female ESCs, REX1 activates Tsix transcription and inhibits Xist transcription [13, 14], thereby blocking initiation of XCI. During differentiation of female ESCs the level of RNF12 is upregulated, resulting in ubiquitination and subsequent proteasomal degradation of REX1 and initiation of XCI by Xist expression. Rnf12 is silenced on the Xi after the onset of XCI, thereby reducing RNF12 levels and preventing onset of XCI on the remaining active X chromosome (Xa). Similarly, the non-coding RNA Jpx is upregulated at the onset of XCI and has been proposed to act as a dosage-sensitive activator of Xist, although a recent report shows that it likely acts in cis [15, 16].

Two recent *Xist* mapping studies show that during the first stage of XCI the X-chromosomal *Xist* spreading is likely to occur by proximity transfer [17, 18]. Although the earliest regions containing enriched occupancies of *Xist* are spread across the entire linear X chromosome, these regions have a high frequency of close contact to the XIC. The early-enriched *Xist* localization sites are gene dense and enriched for silent genes [17, 18]. From these early ‘docking stations’, a second wave of *Xist* spreading occurs by pulling the actively transcribed genes as well as the gene-poor regions in closer proximity to the XIC. *Xist* recruits the Polycomb repressive complex 2 (PRC2) and other proteins involved in gene silencing and chromatin compaction, creating a repressive nuclear compartment present in differentiated cells displaying stable XCI [18–20]. In line with these observations, *Xist* binding is proportional to the increase of PRC2 and the repressive trimethylation of lysine 27 on histone 3 (H3K27me3) on the Xi [18, 21]. Similar to *Xist*, the Polycomb proteins and H3K27me3 are first detected at ~150 canonical sites distributed over the Xi, after which spreading over active genes occurs [21, 22].

Despite recent advances in chromatin-associated changes of the Xi during XCI, little is known on how this affects silencing of genes located on the Xi at the transcript level. Lin et al. [23] investigated gene silencing during XCI by a comparative approach in which differentiating female and male ESCs were profiled in parallel. The female-specific changes were considered to be associated with XCI. However, female and male ESCs maintained in serum-containing media are distinct in their epigenetic make-up, with female ESCs being hypomethylated and male ESCs being hypermethylated [24–26]. Also, differences in activity of the MAPK, Gsk3 and Akt signaling pathways have been reported [27], complicating direct comparisons between ESCs of different sexes.

After establishment of XCI, silencing of the Xi is stably maintained in somatic cells during replication [28]. Although most genes are silent on the Xi at this stage, some genes escape XCI and remain active. In human, at least 15 % of the X-linked genes have been shown to escape XCI [29]. These escape genes are distributed in clusters over the X chromosome [29–31]. This suggests a common regulatory mechanism acting on chromatin domains, the nature of which remains elusive thus far. In mouse, around 15 escape genes have been identified [32–37]. Except for Xist, these genes are generally lower expressed from the Xi compared with the Xa. It has been shown that the escape of Kdm5c in mouse adult tissues is preceded by silencing during early embryonic development [38]. However, for most other escape genes it is currently unclear whether they are initially silenced and reactivated or whether they are never subject to XCI.

Here, we set out to study the dynamics of X-linked gene silencing during the early stages of XCI by differentiation of female ESCs to embryoid bodies (EBs). To avoid comparative analysis between sexes and enable direct quantitative profiling of gene silencing on the Xi, we used female mouse ESCs with non-random XCI and polymorphic X chromosomes [39] to specifically determine the changes occurring on the (future) Xi by high-resolution allele-specific RNA-seq. To investigate later stages, these ESCs were differentiated in vitro to neural progenitor cells (NPCs) [35]. We used allele-specific RNA-seq on the NPCs, in which XCI is fully established and maintained, to correlate the silencing dynamics of genes observed during early XCI with escape from XCI in the NPCs. By associating the genes that escape XCI with topologically associating domains (TADs) as determined in the female ESCs by genome-wide chromosome conformation capture (Hi-C) profiling, we investigate the role of chromatin domains during XCI. By determining the kinetics of gene silencing and correlating this to epigenomic features, our data provide further insight into the formation of the repressive complex during XCI.

## Results

### Experimental setup to study gene silencing on the Xi using allele-specific RNA-seq

**Fig. 1:**
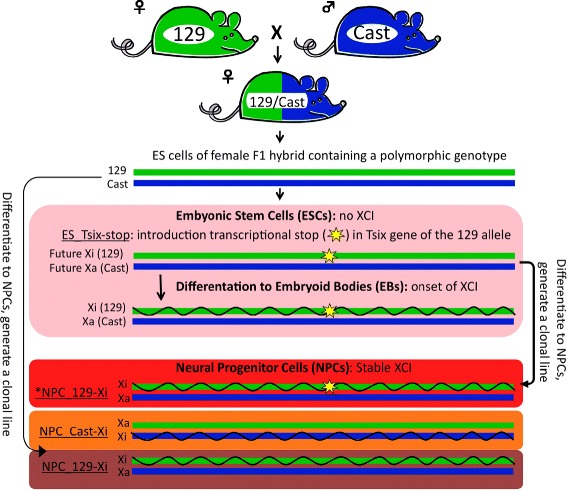
Overview of the setup to study dynamics of gene silencing on the Xi during XCI. Female ES_Tsix-stop ESCs [39] display non-random XCI due to a transcriptional stop in the coding region of Tsix, allowing allele-specific quantification of transcripts originating from the (future) Xi by RNA-seq (*pink background*). To investigate stable XCI from the same female ES_Tsix-stop ESCs, we performed RNA-seq on a clonal NPC line derived from the ES_Tsix-stop ESCs (*NPC_129-Xi, *red background*) [35]. Also, we included RNA-seq on two NPC lines generated from the F1 hybrid ESCs before introduction of the transcriptional Tsix stop. Clonal lines were generated from these two NPC lines to ensure complete XCI skewing towards inactivation of the *M.m. castaneus* (*Cast*)- or the *M.m. musculus* (*129*)-derived X chromosome (NPC_Cast-Xi, *orange background* and NPC_129-Xi, *dark purple background*, respectively) [35]

To determine the dynamics of gene silencing during XCI, we used female ESCs derived from an intercross of *Mus musculus* (*M.m.*) *musculus* 129/SV-Jae (129) and *M.m. castaneus* (Cast) as previously described [39, 40]. Due to the cross of genetically distant mouse strains, this ESC line contains two sets of chromosomes with many polymorphic sites, around 20.8 million genome-wide (~1 single-nucleotide polymorphism (SNP) per 130 bp) and around 0.6 million on chromosome X (~1 SNP per 300; see “Materials and methods”). These sites can be used to perform allele-specific quantification of X-linked and autosomal transcripts by RNA-seq [40]. The introduction of a transcriptional stop signal into the transcribed region of Tsix on the 129-derived X chromosome in the female ESC line results in complete skewing of Xist expression toward the 129-targeted allele [39]. Therefore, the 129-derived X chromosome will always be inactivated during differentiation, allowing specific quantification of transcripts from the Xi and the Xa, respectively (Fig. 1, “ES_Tsix-stop”, pink background). In undifferentiated female ESCs cultured in serum-containing ESC media, inhibition or blocking of Tsix transcription has been shown to be associated with aberrant Xist upregulation and/or partial XCI [6, 23, 41]. Interestingly, we observed a fourfold reduction in *Xist* expression and increased expression of X-linked genes during culturing of the ES_Tsix-stop ESCs in serum-free ESC culture media supplemented with two kinase inhibitors to maintain pluripotency (“2i” ESCs) [24, 27, 42–45] compared with culturing in serum-containing media (“serum” ESCs; Additional file 1: Figure S1). Therefore, we used 2i ES_Tsix-stop ESCs to initiate XCI by differentiation towards EBs and performed allele-specific RNA-seq of the undifferentiated 2i ESCs as well as after 2, 3, 4 and 8 days of EB formation. Validation of the EB time course is documented in Additional file 1: Figure S2, and Figure S3.

To investigate stable XCI, we included allele-specific RNA-seq of three NPC lines that were previously generated in vitro from the polymorphic ESCs [35]. One NPC line was obtained from ESCs after the introduction of the Tsix transcriptional stop (Fig. 1, red), while two NPC lines were obtained from ESCs before the Tsix transcriptional stop was introduced. As the NPCs that do not contain the Tsix transcription stop are heterogeneous with respect to the X chromosome that has been inactivated during random XCI, we generated two clonal NPC lines which showed full skewing of XCI towards the 129- or Cast-derived X chromosome, respectively (Fig. 1, dark purple and orange, respectively) [35]. For comparative purposes we also used a clonal line for the NPCs containing the Tsix transcription stop.

We improved the allele-specific mapping of sequence tags used previously [22] by applying a new procedure based on the GSNAP (Genomic Short-read Nucleotide Alignment Program) algorithm [46], in which the alternative alleles of polymorphic sites are included in the reference genome during mapping. This results in an unbiased mapping of the 129- and Cast-derived sequence tags and an equal contribution in expression from the Cast- and 129-derived genomes in undifferentiated ESCs (Additional file 1: Figure S4a). To enable reliable allele-specific quantification of the RNA-seq, we only included genes for further analysis that (i) showed consistent Cast versus 129 allelic ratios over the polymorphic sites that are present within the gene body (standard error of the mean < 0.1); (ii) contained a total of at least 80 tag counts over polymorphic sites for each allele over the EB formation time course (equivalent to a standard deviation of the allelic ratio of a gene of < 15 % over the time course; see Additional file 1: Figure S4b and “Materials and methods” for further details). Together, our stringent criteria resulted in accurate quantification of allele-specific expression as exemplified in Additional file 1: Figure S4c, d. In total, we obtained allele-specific quantification for 9666 out of a total of 13,909 unique RefSeq genes showing a mean expression of >0.5 RPKM (Reads Per Kilobase of exon per Million mapped reads) over the time course of EB formation (69 %). These include 259 genes on the X chromosome (out of a total of 590 genes with expression > 0.5 RPKM (49 %)). Further details on the samples profiled for this study are provided in Additional file 2: Table S1. Additional file 3: Table S2 contains the gene expression values and allelic counts for all RNA-seq samples.

### XCI during EB formation of female ESCs and in NPCs

**Fig. 2:**
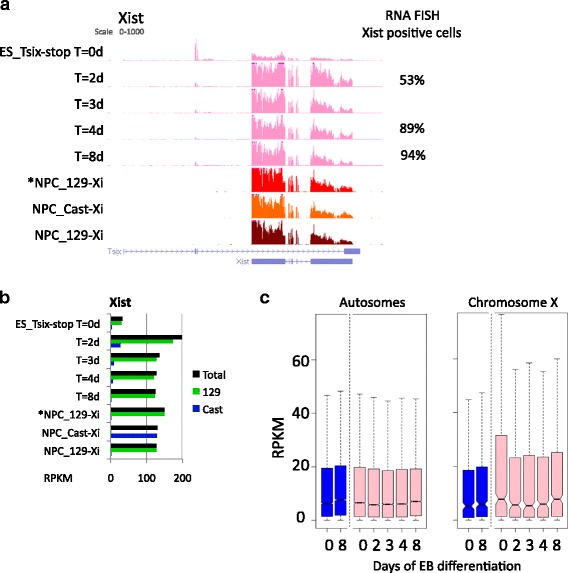
X-linked gene expression during differentiation of ES_Tsix-stop ESCs towards EBs and in NPCs. **a** *Tsix* **/** *Xist* expression dynamics during XCI in ES_Tsix-stop ESCs by EB differentiation, as well as in NPCs. Genome browser view of the Tsix/Xist locus, and the percentage of cells positive for Xist clouds as determined by RNA-FISH. **b** Total Xist expression levels in RPKM (corresponding to (a); in black), as well as the contribution from the 129-derived (*green*) or Cast-derived (*blue*) alleles. **c** Distribution of gene expression in male (E14; *blue*) and female (ES_Tsix-stop; *pink*) ESCs during EB formation. All genes with an expression level of RPKM >0.5 in at least one condition are included (542 and 13,819 genes on the X chromosome and autosomes, respectively)

In order to evaluate the XCI occurring during the EB differentiation of the female 2i ESCs, we examined expression within the XIC. RNA-seq shows Tsix expression in the undifferentiated ESCs (ES_Tsix-stop T = 0 days), while Xist is highly upregulated after two days of differentiation, specifically from the 129 allele (Fig. 2a, b). In line, *Xist* clouds are robustly detected in more than half of the cells after two days of EB formation by RNA fluorescent in situ hybridization (FISH), and in 94 % of the cells after 8 days (Fig. 2a, right column). Activation of *Xist* coincides with a global reduction in expression of X-linked genes of ~30 % after two days of EB formation (Fig. 2c). As the reduction of X-linked expression was not observed during EB differentiation of male cells, nor for autosomal genes, we conclude that this reflects the XCI occurring in the female cells. Within the NPCs, Xist is highly expressed. As expected, Xist is exclusively expressed from the 129 allele in *NPC_129-Xi and NPC_129-Xi, while in NPC_Cast-Xi Xist is expressed from the Cast allele (Fig. 2b). Together, the data show that XCI is robustly initiated on the 129 allele during the EB differentiation time course of ES_Tsix-stop, and stably present in the NPCs.

### Kinetics of gene silencing during XCI on the Xi

**Fig. 3:**
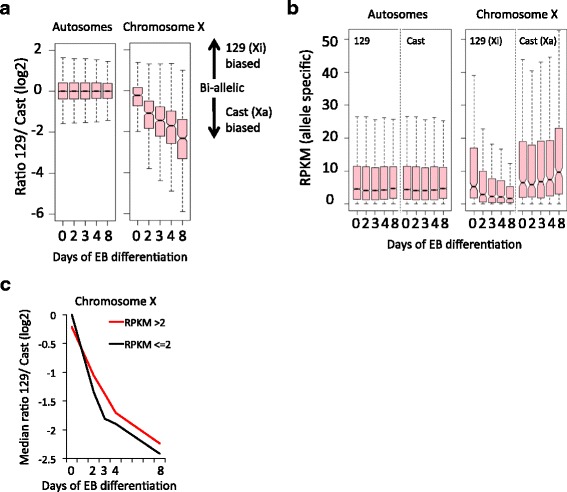
Dynamics of gene silencing on the Xi during XCI using allele-specific RNA-seq. **a** Distribution of relative expression of genes from the 129 versus the Cast allele during EB formation of ES_Tsix-stop. A log2 ratio of 0 represents equal biallelic gene expression from the 129 and Cast alleles, while positive and negative ratios represent higher expression from the 129 or Cast allele, respectively. **b** Distribution of absolute gene expressions from the 129 and Cast alleles (absolute allelic expression values in RPKM; see Materials and methods” for further details) in the ES_Tsix-stop ESCs during EB formation. **c** Median of the relative expression of genes from the 129 versus the Cast allele during EB formation of ES_Tsix-stop for highly and lowly expressed genes on chromosome X (same as the medians shown for the boxplots for chromosome X in Additional file 1: Figure S5b). For highly expressed genes we included genes showing a mean RPKM >2 over the time course (338 genes), while lowly expressed genes showed a mean RPKM ≤2 over the time course (81 genes). See Additional file 1: Figure S5 for further details

To investigate the transcriptional changes occurring on the Xi and Xa specifically, we calculated the ratio of 129/Cast over the time course (Fig. 3a). At a global level, the allelic ratios for autosomal genes remain stable. In contrast, genes on chromosome X show an increasing bias towards expression from the Cast allele, the X chromosome that remains active. After 8 days, gene expression is, on average, approximately fourfold higher from the Xa than from the Xi. Absolute quantification of gene expression shows that expression from the 129 and Cast alleles remains similar on autosomes (Fig. 3b, left panel). For X-linked genes, expression from the 129 allele (Xi) is gradually downregulated, while expression of the Cast allele (Xa) shows a relatively minor but significant (*p* < 0.05 [47]) increase in expression (Fig. 3b, right panel). The increase in activity is not specific for female cells but rather associated with differentiation, as male ESCs also show a similar trend (albeit not significant) of increased X-linked expression during EB formation (Fig. 2c, blue boxplots). Notably, by comparison of the individual time points in female cells we observed a slight but significant difference (*p* < 0.05 [47]) in XCI dynamics between lowly (RPKM ≤2) and highly (RPKM >2) expressed genes, as the lowly expressed genes show faster XCI dynamics than the highly expressed genes (Fig. 3c; Additional file 1: Figure S5).

**Fig. 4:**
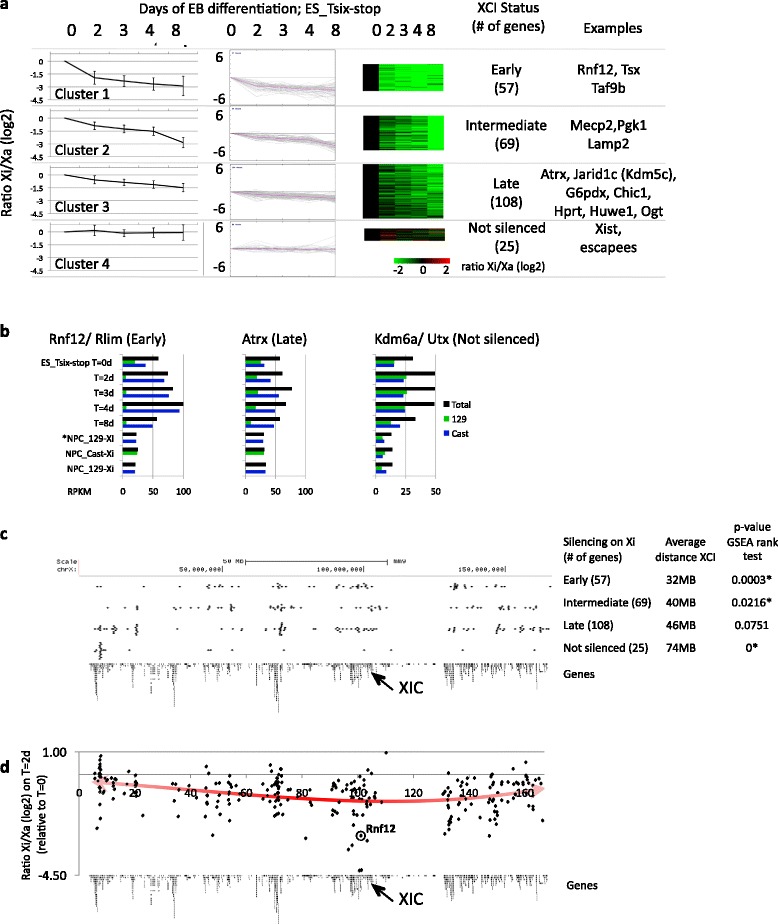
A linear component in the propagation of silencing over chromosome X outwards from the XIC. **a** K-means clustering during XCI identifies four groups (present in the four rows) of genes with different inactivation kinetics on the Xi: early inactivated genes (*top row*), genes that show inactivation at intermediate time points (*second row*), late inactivated genes (*third row*) and genes that are not inactivated (*bottom row*). The first three columns show the inactivation dynamics within the four clusters over the time course as an average (*left*) of the individual genes within the clusters, as a lineplot (*middle*) or as a heatmap (*right*). **b** Examples of genes within the clusters as shown in (**a**). Total expression levels in *black*, the contribution from the 129-derived or Cast-derived alleles in *green* and *blue*, respectively. See Additional file 1: Figure S6 for the genome browser views of the genes. **c** Location of the genes within the clusters as obtained in (**a**) over the linear X chromosome. On the right, the first column shows the clusters and the number of genes within each cluster. The second column shows the average distance of the genes within a cluster to the XIC. The last column shows the *p* value calculated using the gene set enrichment analysis (GSEA) rank test corrected for multiple testing (using FDR (false discovery rate); *significant). The running sum statistics for each cluster for the GSEA is shown in Additional file 1: Figure S9. **d** Early silencing of genes on the Xi plotting the Xi/Xa ratio per gene at day 2 after the onset of EB differentiation over the linear X chromosome. The trend line (polynomial order 3) of the Xi/Xa ratio is plotted in *red*

**Fig. 5:**
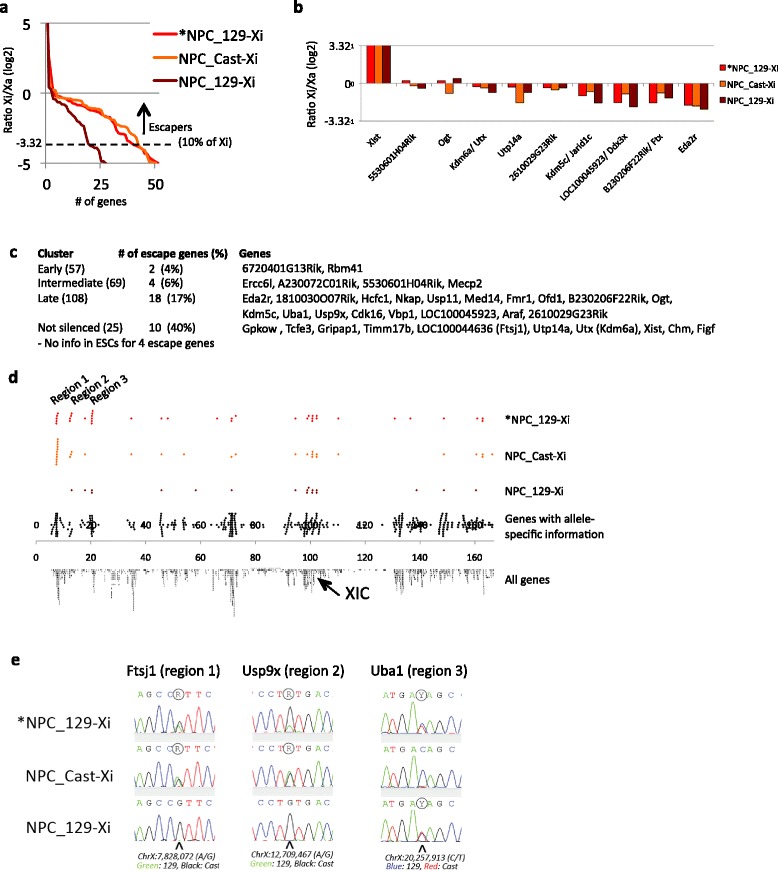
Allele-specific RNA-seq on three NPC lines identifies three distal regions of genes that escape XCI. **a** Ratio of Xi/Xa (*y-axis*; for each of the three NPC lines sorted from highest to lowest) for genes showing a log2 ratio of at least −5. We set the cutoff for escape on 10 % relative expression from the Xi versus the Xa (log 2 ratio of > −3.32; similar to Yang et al. [37]). **b** Xi/Xa ratio of genes that escape XCI in all three NPC lines. **c** Distribution of the escape genes identified in *NPC_129-Xi over the four clusters as characterized in Fig. 4a. **d** Localization of the escape genes within each NPC line over the linear X chromosome (see also Table 1). The *black dots* on the *fourth row* represent all X-linked genes for which high-confidence allele-specific ratios were obtained in NPCs. **e** Validation of the escape genes within the three escape regions by Sanger sequencing of cDNA. See Additional file 1: Figure S13 for the full panel of 13 genes that we validated, and for further details

To further stratify genes showing similar XCI dynamics, we performed K-means clustering on the Xi/Xa ratio over the time course (Fig. 4a). The clustering revealed four clusters containing genes that show similar dynamics. The genes in cluster 1 are mainly silenced on the Xi within 2 days of EB formation, and therefore these genes are inactivated relatively fast (labeled as “early”). The genes in cluster 2 (labeled as “intermediate”) mainly show silencing between 4 and 8 days of EB formation. Genes in cluster 3 show some initial silencing of the Xi over the time course, and only show a mild bias for higher expression from the Xa at the latest time point of 8 days EB formation. However, most of the cluster 3 genes are fully silenced during stable XCI, including in NPCs (as discussed later; Fig. 5). Therefore, we labeled this cluster as “late”. The relatively small number of genes present in cluster 4 did not show any sign of silencing (labeled “not silenced”), and include many known escape genes such as Xist, Kdm6a (Utx), Utp14a and Chm. Figure 4b shows three examples of genes present in the “early”, “late” and “not silenced” cluster, respectively. Genes within the “late” cluster were significantly higher expressed than genes in the other clusters (Additional file 1: Figure S7) [47], reinforcing the observation that highly expressed genes generally show slower silencing kinetics during XCI (Fig. 3c; Additional file 1: Figure S5).

A comparison of the kinetic clusters with a previous study that used RNA FISH to determine X-linked silencing at the single gene level [19] shows that Mecp2, Pgk1 and Lamp2 (present in the “intermediate” cluster 2 in our study (Fig. 4a)) are robustly inactivated in both studies. Atrx, Jarid1c (Kdm5c) and G6pdx show late silencing by RNA FISH as well as by the allele-specific RNA-seq (“late” cluster 3; Fig. 4a). Only Chic1 shows different inactivation kinetics, being early inactivated by RNA FISH, while here it is present in the “late” cluster 3 (Fig. 4a). Altogether, the high overlap with the RNA FISH validates the clusters obtained for gene silencing on the Xi by the allele-specific RNA-seq.

During a comparative approach of female and male ESCs to identify female-specific changes associated with XCI, Lin et al. [23] characterized four gene clusters each showing different kinetics of X-linked gene silencing. In terms of kinetics, these clusters resemble the clusters as identified in Fig. 4a. However, the genes within the clusters obtained by this comparative approach show poor overlap with the respective clusters obtained in the current study (Additional file 1: Figure S8 and Additional file 4: Table S3). This might well be caused by the differences in the epigenetic make-up [24–26] and the differences in activity of the MAPK, Gsk3 and Akt signaling pathways [27] between male and female ESCs, resulting in a significant delay in differentiation of female ESCs relative to male ESCs [27]. Being independent of comparative analysis with male ESCs, the use of allele-specific RNA-seq circumvents these issues and possible confounding effects.

### Spreading of gene silencing over the X chromosome

We next plotted the genes present in the four clusters over the linear X chromosome (Fig. 4c). Interestingly, genes of the “early” cluster are, on average, closer to the XIC than genes within the other clusters. Genes in the “intermediate” cluster are again closer to the XIC than genes in the “late” and “not silenced” clusters. A relatively high number of genes of the “late” and “not silenced” clusters are located at sites very distal from the XIC. A gene set enrichment analysis (GSEA) rank test (Fig. 4c) reveals the significant correlation between the distribution of genes within the “early”, “intermediate” and “not silenced” clusters and their distance to the XIC, and recapitulates the observed distributions of the clusters over the X chromosome (Additional file 1: Figure S9).

Around half of the silencing on the Xi (on average 46 % per gene) occurs during the first two days of EB formation. To further study the changes occurring at the early stages, we plotted the Xi/Xa ratio of the 256 genes at T = 2 days relative to T = 0, and fitted a trend line (Fig. 4d). At this early time point, genes proximal to the XIC show more silencing on the Xi compared with distal genes. Additionally, the top five most silenced genes on the Xi (Tsx, C77370, Pja1, Dlg3 & Taf9b) are all within 5 Mb of the XIC (Fig. 4d). Plotting of the other time points relative to the undifferentiated ESCs shows a subsequent spread over the X chromosome, with the exception of the very distal region around 10 Mb (Additional file 1: Figure S10). This region, which contains many genes (16 out of 25) of the “not silenced” cluster (Fig. 4c), is further discussed in the next paragraph.

Together, the silencing dynamics of X-linked genes shows that there is a linear component of XCI during gene silencing over chromosome X. Interestingly, Rnf12 (Rlim) is silenced early (in cluster 1; Fig. 4a, b), and shows one of the highest Xi/Xa ratio of all genes (Fig. 4d). Globally, Rnf12 shows a modest but rapid upregulation at very early time points (between 2 and 4 days of EB formation; Fig. 4b; Additional file 1: Figure S6). Soon after this initial increase, Rnf12 is downregulated and becomes stably silenced on the Xi (as shown below in the NPCs). The observed Rnf12 dynamics is in line with its proposed function as a dose-dependent XCI activator [10, 13, 16], which is silenced early to prevent initiation of XCI on the second allele. Jpx (2010000I03Rik), the other gene implicated in activation of Xist during XCI [15, 36], is also rapidly upregulated at the onset of XCI. However, Jpx remains at an elevated level after the initial upregulation (Additional file 3: Table S2). Jpx remains active from the Xi during EB formation as signals over the polymorphic sites of Jpx are equally distributed over the Xi and Xa, albeit at low coverage (Additional file 2: Table S2). Also, Jpx escapes XCI in the NPCs (as shown below). Different from Rnf12, it is likely, therefore, that (transcription of) Jpx is required for continuous activation of Xist on the Xi at all stages of Xist-mediated XCI.

A previous paper reported the presence of a subset of genes near the XIC that are silenced in undifferentiated serum ESCs due to initiating XCI [23]. Although we detect 12 genes on the X chromosome that show allelic bias in the undifferentiated ESCs, these are not consistent with respect to the allele that is expressed (seven genes show higher expression from the future Xi, five from the future Xa) and their location is evenly distributed over the linear X chromosome (Additional file 1: Figure S11). This reinforces the conclusion that we do not observe any signs of initiating XCI in the undifferentiated 2i female ES_Tsix-stop ESCs used for the current study.

### Escape genes on the Xi in the NPCs

To evaluate the XCI status of the four kinetic clusters during stable XCI, we performed allele-specific RNA-seq on a NPC line generated from the ES_Tsix-stop ESCs, as well as from two NPC lines generated from the same ESCs before the Tsix stop mutation was introduced (Fig. 1). As expected for the stably inactivated X chromosome, we did not observe any signal from the Xi in the NPCs for a large number of X-linked genes (0 sequence tags for ~70 % of the genes for which allelic information is present; Additional file 5: Table S4), while robust expression was detected from the Xa. Plotting of the Xi/Xa ratio shows that only a limited number of genes show a >10 % contribution in expression from the Xi relative to the Xa (Fig. 5a), which previously was applied as main classifier to call genes escaping from XCI [37]. Only Xist is expressed higher from the Xi compared with the Xa, while four other genes (5530601H04Rik, Ogt, Kdm6a (Utx) and 2610029G23Rik) show roughly equal expression from the Xi and the Xa in all three NPC lines (Fig. 5b). The remaining genes show (much) lower or no contribution of expression from the Xi (Fig. 5a, b). In total 38, 34 and 18 genes escape XCI in the *NPC_129-Xi, NPC_Cast-Xi and NPC_129-Xi lines, respectively (Fig. 5a; Table 1). Besides six genes that had too little or no coverage over polymorphic sites in our dataset, almost all escapers previously identified in mouse by Yang et al. [37] (in embryonic kidney-derived Patski cells), Splinter et al. [35] (in NPCs) and Li et al. [33] (in neural stem cells) are escaping XCI in at least one NPC line. Only Shroom4 and Car5 are stably inactivated in the NPCs used for the current study, while they escape XCI in the Patski cells as reported by Yang et al. [37] (see Table 1 for detailed comparisons). Most genes that escape XCI in mouse brain tissue [48] also escape XCI in NPCs (Table 1). In line with their tissue-specificity, only one gene (Utp14a) of the 24 genes that specifically escape XCI in mouse spleen and/or ovary [48] escapes XCI in the NPCs. Furthermore, nearly all genes that escape in mouse trophoblast cells during imprinted XCI [49] (and for which there is sufficient allele-specific coverage in the NPCs profiled in the current study) escape XCI in at least one of the NPC lines (Table 1). However, we identify more escape genes compared with these previous studies (Table 1), as further discussed below.

**Table 1:** Genes escaping XCI in any of the three NPC lines in comparison with other studies

Gene	S	Mb start position	*NPC_ 129-Xi	NPC_ Cast-Xi	NPC_ 129-Xi	[37]	[35]	[33]	[48]	[49]	Other	[29] (human)	Cluster
Kdm6a/Utx^a^	+	17.7	**−0.3**	**−0.4**	**−0.8**	*	*	*	*	*		**UTX/DUSP21 (9/9)**	4
Utp14a	+	45.6	**−0.3**	**−1.7**	**−0.8**			*		*		UTP14A (3/9)	4
Eda2r	-	94.6	**−1.9**	**−2.0**	**−2.3**							XEDAR (4/9)	3
Ogt	+	98.8	**0.2**	**−0.9**	**0.4**		*			*		OGT (0/9)	3
Xist	-	100.7	**8.7**	**6.5**	**10.2**	*	*	*	*	*		**XIST (9/9)**	4
Ftx/ B230206F22Rik	-	100.8	**−1.7**	**−0.8**	**−1.3**				*	*	* [32]	NA	3
5530601H04Rik	-	102.3	**0.2**	**−0.2**	**−0.4**				*	*	* [34]	NA	2
2610029G23Rik	+	102.3	**−0.4**	**−0.6**	**−0.4**	*	*	*		*	* [34]	NA	3
Kdm5c/Jarid1c	+	148.7	**−1.1**	**−0.7**	**−1.7**	*	*	*	*	*		NA	3
Nkap	+	34.7	**−0.6**	**−0.3**	−8.2		*			*		NKAP (1/9)	3
Hcfc1	-	71.2	**−2.5**	**−2.9**	-		*					**HCFC (6/9)**	3
Vbp1	+	72.8	**−2.3**	**−3.0**	-		*					VBP1 (1/9)	3
Jpx/2010000I03Rik/Enox	+	100.7	**−1.1**	**−1.3**	***NA*** ^b^					*	* [36]	NA	NA
Chm	-	110.3	**0.2**	**−2.9**	***−3.0***		*					**CHM (6/9)**	4
Gpm6b	+	162.8	**−0.4**	**−0.9**	−3.6				*	*		**GPM6B (8/9)**	NA
Ofd1	-	162.9	**−3.2**	**−0.2**	−7.5							**OFD1 (6/6)**	3
Mecp2	-	71.3	**−1.8**	-	**−2.3**							MECP2 (0/9)	2
Ercc6l	-	99.4	**−0.4**	-	**−0.6**							FLJ20105 (2/9)	2
Ndufb11	-	20.2	**−0.5**	-	-							P17.3 (0/9)	NA
A230072C01Rik	+	20.5	**−2.9**	-	-							NA	2
6720401G13Rik	-	48.0	**−2.8**	-	−5.4	*	*					NA	1
Fmr1	+	65.9	**−2.4**	-	-		*					FMR1 (1/9)	3
Trmt2b	-	130.8	**−0.5**	-	−3.9							FLJ12687 (0/9)	NA
Rbm41	-	136.5	**−2.4**	-	−5.8							FLJ11016 (3/9)	1
Figf	+	160.9	**−2.2**	−3.8	-							NA	4
Mmgt1	-	53.9	-	**−0.5**	-							NA	
Siah1b	-	160.5	-	**−1.6**	−5.4					*		NA	
Mid1	+	166.3	−4.9	**−0.4**	−7.1	*	*			*		MID1 (1/9)	
Sox3	-	58.1	-		**0.3**							NA	
Taf1	+	98.7	***−1.1***	***−2.9***	**−0.6**		*			*		TAF1 (0/5)	
Gm5643/Hnrnpa1l2	+	138.7	***−1.2***	***2.6***	**−1.6**							NA	
Zrsr2	-	160.4	-	−5.9	**−2.0**					*		NA	
**Region 1**													
Gpkow^a^	+	7.27	**−2.4**	**−1.2**	−8.4							GPKOW (0/9)	4
Wdr45	+	7.30	***−3.0***	**0.0**	-							WDRX1 (0/9)	
Tcfe3	+	7.34	**−1.9**	**−0.8**	−6.7							TFE3 (0/9)	4
Gripap1^a^	+	7.37	**−1.1**	**−1.5**	−6.3					*		GRIPAP1 (1/9)	4
Slc35a2	+	7.46	***−1.6***	**1.3**	-					*		SLC35A2 (0/9)	
Timm17b	+	7.48	**−0.9**	**−1.1**	-							TIMM17B (0/9)	4
Ftsj1/LOC100044636^a^	-	7.83	**−0.3**	**−1.2**	-							FTSJ1 (0/9)	4
Hdac6	-	7.52	−7.5	**−1.6**	-					*		HDAC6 (0/5)	
Wdr13^a^	-	7.71	-	**−1.0**	-							WDR13 (0/9)	
Rbm3^a^	-	7.72	−6.4	**−1.7**	−5.8							RBM3 (0/9)	
2900002K06Rik^a^	+	7.72	-	**−0.2**	-							NA	
Ebp	-	7.77	-	**−2.6**	-							EBP (0/9)	
**Region 2**													
Ddx3x/LOC100045923^a^	+	12.86	**−1.7**	**−0.9**	**−2.1**	*	*	*	*			**DDX3X (9/9)**	3
Med14^a^	-	12.34	**−0.5**	**−1.4**	−7.3							**CRSP2 (6/6)**	3
Usp9x^a^	+	12.65	**−0.9**	**−1.3**	−9.0		*					**USP9X (9/9)**	3
1810030O07Rik^a^	-	12.25	**1.3**	−5.1	−5.9	*	*					**MGC39350 (8/9)**	3
**Region 3**													
Uba1^a^	+	20.24	**−0.4**	−3.7	**−0.9**							**UBE1 (9/9)**	3
Cdk16/Pctk1^a^	+	20.27	**−1.1**	-	**−0.4**		*					**PCTK1 (7/7)**	3
Usp11^a^	+	20.28	**−0.5**	−7.1	−8.2							**USP11 (4/9)**	3
Araf	+	20.43	**−1.0**	-	-							ARAF1 (0/6)	3

Data in the columns *NPC_129-Xi, NPC_ Cast-Xi, NPC_ 129-Xi are represented as ratio Xi/Xa (log2). The bold ratios indicate escape from the corresponding gene, the bold italic ratios indicate that too little polymorphic sites and/or reads from Xi were present to classify the gene as escaper. A dash or values < −3.32 in these columns indicate that the corresponding gene is subject to XCI. Genes that escape XCI in human fibroblasts are in bold in the second-last column, with the ratio indicating the number of clones showing escape as observed by Carrel and Willard [29]. For the study by Berletch et al. [48], we only included escape genes from the mouse brain, while spleen and ovary are not included in this table. Cluster numbers in the last column refer to Fig. 4a and are indicated for genes that escape XCI in *NPC_129-Xi. *S* strand. ^a^Escape is validated by RT-PCR sequencing (Additional file 1: Figure S13). ^b^Only two tags from the Xi, no signal from Xa. *Identified as escaper in other studies

Comparison of the kinetic clusters (Fig. 4) with the 38 genes that escape XCI in *NPC_129-Xi (obtained by differentiation of the ES_Tsix-stop ESCs) shows that the majority of escape genes (28 genes in total) are present in the “late” and “not silenced” cluster (Fig. 5c; Table 1). Only six genes are present in the earlier clusters (four escape genes were not included in the clustering due to insufficient coverage of the polymorphic sites) in the ESCs. The “not silenced” cluster shows the highest enrichment of escape genes (40 %; Fig. 5c). Therefore, the escape genes seem to be (partly) excluded from XCI from a very early point onwards. The silencing of escape genes that are present in the “late” cluster, such as Ogt, Jarid1c (Kdm5c) and Ftx, might indicate that these genes are initially silenced, after which they are reactivated, as has been shown for Jarid1c (Kdm5c) [38, 50]. However, EBs are complex mixtures of cells of which only part is ectoderm or reflect intermediate stages towards NPC formation. Therefore, the silencing observed in the “late” cluster for genes that escape XCI in NPCs might as well originate from cells within the EBs other than ectoderm cells or cells differentiating towards NPCs.

To further investigate the remarkable difference in the number of genes escaping XCI in the three NPC lines (Fig. 5a, Table 1), we plotted the genes escaping XCI over the linear X chromosome (Fig. 5d). This shows that all three NPCs share escape genes over most of the X chromosome, except for three distal regions (regions 1–3) that were also pronounced in cluster 4 within the previous analysis (Fig. 4c, “not silenced”). Within these regions *NPC_129-Xi and NPC_Cast-Xi, but not NPC_129-Xi, show a contiguous number of three or more genes that escape XCI, while genes that are subject to XCI are absent in these regions (Fig. 5d; see Table 1 for the genes present within the escape regions). Escape region 3 is specific to *NPC_129-Xi, while escape regions 1 and 2 are largely shared by *NPC_129-Xi and NPC_Cast-Xi, with region 1 containing more escape genes in NPC_Cast-Xi compared with *NPC_129-Xi (Fig. 5d; Additional file 1: Figure S12a). Sanger sequencing of cDNA from the three NPC lines confirmed the pattern of escape from XCI in the three regions for nearly all genes tested (6, 4 and 3 genes for regions 1, 2 and 3, respectively; Fig. 5e; Additional file 1: Figure S13; Table 1). The only discrepancy concerns 1810030O07Rik, which, in contrast to the RNA-seq results (Table 1), shows escape from XCI in NPC_Cast-Xi using cDNA Sanger sequencing (albeit at a low level; Additional file 1: Figure S13). This would be in line with other genes in region 2, which also escape XCI in NPC_Cast-Xi as well as in *NPC_129-Xi. Interestingly, the escape is also reflected in the total expression levels of the genes: the escape genes within region 1 are significantly higher expressed in the two lines in which they escape compared with NPC_129-Xi, in which they are silenced on the Xi (Additional file 1: Figure S12b; *p* < 0.05 [47]).

### Stability of the three XCI escape regions in the NPCs

In light of the differences in escape regions between the three different NPC lines, we next considered the stability of the escape genes during cell culture. We cultured the three NPC lines for one month (more than ten passages) and performed allele-specific RNA-seq to assess the genes escaping XCI. The escape genes identified in the three NPC lines showed a large overlap with the escape genes as determined at the start of the culturing (Additional file 1: Figure S14a), including the escape genes present in the three escape regions (Additional file 1: Figure S14b). Notably, most genes showing differential escape before and after one month of NPC culturing are expressed from the Xi at a relative level of ~10 % compared with the Xa and just did not make the cutoff in one condition (data not shown). Together, we conclude that the genes escaping XCI in the NPCs are stably maintained over time.

### Regions of genes that escape XCI in the NPCs are associated with TADs

**Fig. 6:**
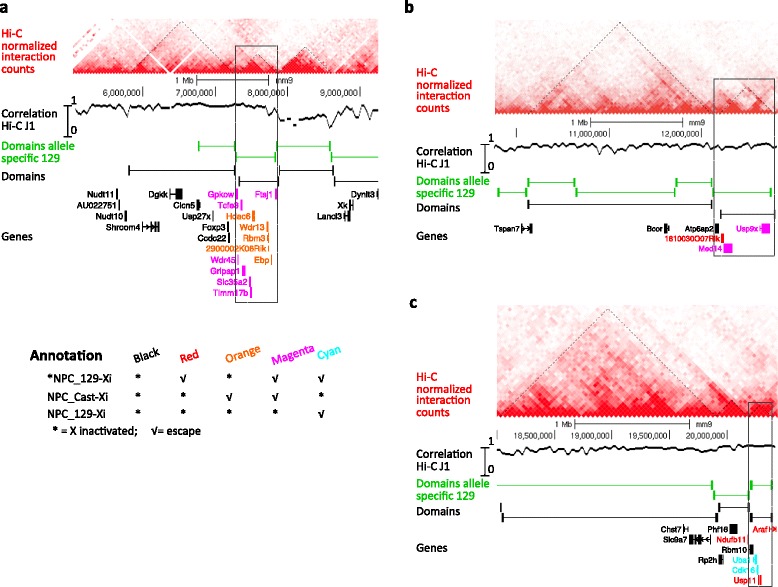
The three regions escaping XCI colocalize with TADs as identified in ES_Tsix-stop ESCs. **a**–**c** Overview of the TADs present at regions 1, 2 and 3 [indicated with a box in (**a**), (**b**) and (**c**), respectively] in the female ES_Tsix-stop ESCs. In *red*, the interaction matrix used for TAD calling with domains indicated by *dashed lines*. The *second row* shows the spearman correlation between the 40 kb-binned Hi-C interaction matrices of the female ES_Tsix-stop and male J1 ESCs [51] (see “Materials and methods” for further details). The legend for genes that escape XCI or genes that are silenced is indicated in (**a**). Coloring of genes indicates escape in one or two NPC lines, respectively, while genes in *black* are X-inactivated in all NPC lines. Wdr45 and Slc35a2 are included as escape gene for *NPC_129-Xi as the contribution in gene expression from the Xi is >10 % (Table 1). Additional file 1: Figure S17 contains the same information as Fig. 6, but includes genes for which no allelic information was obtained (mainly due to low expression or the absence of polymorphic sites), as well as the interaction matrix in male J1 ESCs obtained from Dixon et al. [51] for comparison

The clustering of genes that escape XCI, as observed in the NPCs, might suggest regulatory control at the level of chromatin domains in which epigenetic domains on the Xi are affected during inactivation. To further investigate the chromatin conformation of the three escape regions, we determined the TADs in the undifferentiated ES_Tsix-stop ESCs using Hi-C profiling (Additional file 6: Table S5 and Additional file 1: Figure S15). The TADs of the female ES_Tsix-stop show a very high overlap with the TADs previously identified in male J1 ESCs [51], on autosomes as well as on chromosome X (Additional file 1: Figure S15c, correlation track; Additional file 1: Figure S16). Overlaying the three escape regions as identified in the NPCs with the Hi-C profile shows that the genes within escape regions coincide within individual topological domains (Fig. 6a–c; Additional file 1: Figure S17a–c). Also, the three domains associated with the escape regions do almost exclusively contain genes that escape from XCI. The exceptions involve Ddx3x, which is part of escape region 2 but located in a TAD neighboring the TAD associated with region 2 (not shown in Fig. 6b), as well as Atp6ap2 and Rbm10, which are subject to XCI but present within the TADs associated with regions 2 and 3, respectively (Fig. 6b, c). However, Atp6ap2 and Rbm10 are elocalized at the boundaries of the TADs associated with regions 2 and 3, respectively, and have their upstream promoter regions in the neighboring TADs, which might explain their silencing. The topological domains neighboring the three escape regions, but also on the remainder of the proximal part of chromosome X, do hardly contain escape genes but rather genes that are subject to XCI on the Xi (Figs. 5d and 6a–c). Interestingly the 10 kb promoter region of Ndufb11, positioned outside but in close proximity to escape region 3, is located within the TAD associated with region 3 (Fig. 6c). This might explain the escape we observe for Ndufb11.

To determine the TADs over the three escape regions on the 129-derived X chromosome (that is being inactivated during differentiation of the ES_Tsix-stop ESCs), we performed allele-specific calling of TADs. In line with the non-discriminative 129/Cast Hi-C analysis, the allele-specific Hi-C shows the presence of the domains overlaying the regions of escape on the 129-derived X chromosome (Fig. 6a–c). For validation of the overlap between the escape regions and the TADs, we analyzed allele-specific RNA-seq data from very similar 129/Cast hybrid female NPCs generated by Gendrel et al. [52]. We observed a high number of escape genes within the three regions (Additional file 1: Figure S18), but not in neighboring regions/domains, showing that the three regions have a consistent tendency to escape XCI in NPCs. Together, these observations suggest that the three regions escaping XCI represent TADs that are affected during the onset of XCI.

To further investigate the spatial organization of the three escape regions within the NPCs, we overlaid these regions with the allele-specific chromosome conformation capture-on-chip (4C) profiles generated by Splinter et al. [35] on the same NPC lines as analyzed in the current study. This showed that the three escape regions in the NPCs represent three domains that are clustered together in nuclear space with other genes that escape XCI within the NPCs (data not shown).

### Association of escape clusters with TADs in human

In human 15 % of the X-linked genes escape XCI as assayed in hybrid fibroblast lines [29]. Most of these escape genes are present in the short arm (Xp) of the X chromosome, where they are present in clusters. To assess whether in human these clusters as identified by Carrel and Willard [29] correlate with TADs, we overlaid the escape clusters with TADs determined in human female fibroblasts by Dixon et al. [51] (Additional file 1: Figure S19). For 15 of the 17 TADs, all the associated genes within the respective TAD either escape XCI or are silenced (Additional file 1: Figure S19a). The TADs escaping XCI and the silenced TADs show an alternating pattern over the X chromosome (Additional file 1: Figure S19b). Therefore, the control of these clustered escape genes in human might well occur at the level of the TADs, in line with our observations in mouse NPCs.

## Discussion

In this study, we determined the dynamics of gene silencing on the (future) Xi by allele-specific RNA-seq during differentiation of female ESCs. We optimized the allele-specific RNA-seq mapping by GSNAP [46] in an efficient and straightforward procedure, thereby obtaining unbiased high-resolution gene expression profiles from both alleles. The silencing kinetics for individual genes during XCI reveals a linear component in the propagation of inactivation over the Xi. This is supported by the increase in distance of four kinetic clusters associated with gene silencing, as well as by the high ratio of gene silencing for genes near the XIC at very early stages of XCI. The escape from XCI of three regions very distal from the XIC, in differentiated ES_Tsix-stop ESCs as well as in NPCs, might be a consequence of incomplete linear spread. It has been shown that XCI-mediated silencing can only occur in a short time-window of embryonic development/differentiation also referred to as the “window of opportunity” [53]. As a consequence, cells that do not complete XCI within this time frame might fail to inactivate parts of the X chromosome that are at greater distance from the XIC and hence silenced late. The NPCs used in the current study, as well as the NPCs generated by Gendrel et al. [52] in which the escape regions are also present, have been derived from ES_Tsix-stop ESCs [35]. During the extensive in vitro differentiation towards NPCs, a subset of ESCs might have completed XCI (NPC_129-Xi), while in other cells the XCI process remains incomplete (*NPC_129-Xi and NPC_Cast-Xi). In the latter cells, parts of the Xi remain active, as they are not silenced during the window of opportunity. Apparently, the activity of the non-silenced genes on the Xi is tolerated in the NPCs, although it might affect cell viability as we noticed that the *NPC_129-Xi and NPC_Cast-Xi NPC lines show increased doubling times compared with NPC_129-Xi.

If indeed the escape regions result from incomplete XCI during the window of opportunity, their localization at regions very distal to the XCI would further support a linear model of propagation of XCI from the XIC over the (future) Xi. However, similar to what has been shown for imprinted XCI of the paternal Xi during early mouse development [54], linearity clearly only explains part of the silencing dynamics we observe. Various genes near the XIC are inactivated late and show no signs of silencing at early time points, while other genes very distal from the XIC are silenced early. Therefore, other components such as spatial organization of the X chromosome, TADs (as discussed below) and local chromatin environment likely play important roles in the silencing dynamics on the Xi. Indeed, it has been shown that the earliest regions containing enriched occupancy of *Xist* are spread across the entire linear X chromosome, but do have spatial proximity to the XIC [17, 18]. Furthermore, also the level of gene expression affects the kinetics of XCI silencing, as we observe that highly expressed genes show a slight but significant delay in silencing compared with lowly expressed genes. This might be caused by the fact that it takes longer for these highly expressed genes to alter the local chromatin environment by depositing marks associated with silencing, such as H3K27me3 [22, 55, 56]. On the other hand, the stability of the various RNAs also influences the kinetics of X-linked silencing during XCI. Stable RNAs have a longer half-life and will, therefore, show slower silencing dynamics in our analysis. A recent study investigating stability of X-linked transcripts showed an overall increase in half-life of X-linked transcripts versus autosomal transcripts [57, 58]. Amongst X-linked transcripts, the half-life varied between 2 and 15 h, with the median half-life being 6 h. Since this time frame is much shorter than the 8-day course of EB differentiation, stability of RNA likely has little influence on the clustering we performed (Fig. 4). Rather, the clustering has been dictated by silencing of transcription on the chromatin.

The three escape regions identified in the current study (Figs. 5 and 6) largely correspond to TADs as characterized in the undifferentiated female ESCs. Together with the observation that the escape clusters in human closely correlate with TADs, this suggests a functional role for the TADs during XCI. Previously, TADs have been implicated in the regulation of XCI within the XIC, with the promoters of Tsix and Xist being present in neighboring TADs with opposite transcriptional fates [59]. Furthermore, it has been shown that TADs align with coordinately regulated gene clusters [59]. The current observation that the regions escaping XCI correspond to TADs suggests that genes within TADs are co-regulated to induce silencing in a domain-type fashion during XCI. This would imply that TADs are the functional compartments in the higher order chromatin structure that are targeted for inactivation during initiation of XCI. Once targeted, silencing might be propagated within the TAD such that the associated genes become inactivated. How this would work remains to be resolved, but the functional mechanisms might resemble those acting in long range epigenetic silencing (LRES) by which large regions (up to megabases) of chromosomes can be co-coordinately suppressed [60].

Together, the dynamics of XCI we observe fit with previously proposed biphasic models in which secondary spread of inactivation occurs via so-called relay elements, way stations or docking stations, the nature of which still remains elusive [18, 21, 22, 61] (see Ng et al. [62] for a recent review). Our study suggests that TADs are the primary targets during propagation of XCI, after which secondary spread occurs within TADs. Such involvement of TADs in XCI is likely to be very early during the inactivation process, as it has been shown that the Xi has a more random chromosomal organization at later stages in which global organization in TADs is reduced and specific long-range contacts within TADs are lost [35, 59, 63]. An interesting possibility to further investigate the role of TADs during inactivation of the (future) Xi is to investigate gene silencing within TADs during XCI — for example, during the EB formation time course we performed. However, the current resolution of allele-specific RNA-seq lacks resolution for such analysis, mainly due to (i) the limited number of polymorphic sites available to distinguish both alleles; and (ii) the very high depth of sequencing necessary to obtain reliable allele specific calls for lowly expressed genes (which by definition will have low coverage over polymorphic sites). For the current study we obtained allelic information for 259 X-linked genes over the EB differentiation time course, while the X chromosomes consists of 124 TADs (Additional file 7: Table S5). This average number of genes per TAD is insufficient to study expression dynamics within TADs.

Besides the genes within the escape regions, none of the remaining genes on the X chromosome are present in clusters of contiguous escape genes. Also, other escape genes co-occupy the TAD in which they are localized with genes that are subject to XCI. Therefore, the escape of genes outside the escape regions is likely instructed by epigenetic features other than TADs. This might also be the case for the well-known escape gene Ddx3x, which is part of escape region 2 but not part of the TAD that is associated with this region. Next to the escape genes reported in Table 1, we detect some (very) low level escape in all three NPC lines: an additional ~50 genes show <10 % contribution of the Xi to the total expression of a gene (in most cases <1 %) mostly corresponding to five or less sequence tags (Additional file 5: Table S4). A recent study reporting a similar finding in NPCs proposed that this is associated with a relaxation in the epigenetic state in NPCs as well as in neural stem cells in brain tissue [52], suggesting that reactivation from the Xi can occur for these genes. Also for individual escape genes such as Kdm5c, it has been reported that they were initially silenced at the onset of XCI, after which they are reactivated later during development from the Xi [38, 50]. However, the majority of escape genes in the NPCs identified in the current study already (largely) escape silencing during establishment of XCI, as they are present in the “late” or “not silenced” kinetic clusters 3 or 4 in the female EB differentiations. This suggests that escape genes are already excluded from XCI from the start, and that most of these escape genes, therefore, likely contain (epi)genetic features that exclude them from being silenced during propagation of XCI.

By determining global levels of gene expression at different stages of differentiation and development, our data furthermore provide insight into the dynamics of dosage compensation between the X chromosome and autosomes. In ESCs, the mean level of X-linked gene expression in female and male is 1.50- and 0.86-fold higher, respectively, than expression from autosomal genes (Additional file 1: Figure S1; Fig. 2c). Compared with ESCs, expression of female X-linked genes in epiblast stem cells (EpiSCs) is reduced, while expression of male X-linked genes is increased. Autosomal expression is relatively stable between female and male ESCs and EpiSCs. This results in very similar levels of expression between autosomal and X-linked genes in male and female EpiSCs (Additional file 1: Figure S1), in line with previous observations by Lin et al. [23]. Very similar dynamics are obtained during EB differentiation, during which X-linked genes are slightly upregulated from the Xa in female (Fig. 2c) and the single X chromosome in male ESCs (Fig. 3b, right panel). This suggests that full dosage compensation in differentiated cell types is achieved by upregulation of the genes on the Xa in female and the single X chromosome in male cells during early embryonic development.

## Conclusions

Our study provides the first comprehensive allele-specific analysis of gene silencing during XCI. It shows that a linear model can partly explain propagation of silencing over the X chromosome, while also the level of expression affects gene silencing. Given the overlap between regions of XCI escape and TADs in the mouse NPCs, as well as in human fibroblasts, we hypothesize that X-linked TADs function as modular domain structures that are being targeted in primary propagation of silencing. After this initial targeting, secondary spread of XCI might occur within the TADs. During this process, gene expression of the Xa is upregulated, resulting in complete dosage compensation between X-linked and autosomal genes in differentiated cell types. The molecular mechanisms by which this upregulation occur are currently unclear, but might involve transcriptional as well as posttranscriptional regulatory mechanisms.

## Materials and methods

### Cells and cell culture

ESCs were cultured without feeders in the presence of leukemia inhibitory factor (LIF, 1000 U ml^−1^) either in Glasgow modification of Eagles medium (GMEM) containing 10 % fetal calf serum (called “serum” medium), or in serum-free N2B27 supplemented with MEK inhibitor PD0325901 (1 μM), GSK3 inhibitor CH99021 (3 μM), penicillin (100 U ml^−1^), streptomycin (100 mg ml^−1^), glutamine (1 mM), non-essential amino acids (0.1 mM) and β-mercaptoethanol (0.1 mM) (together called “2i” medium) [45]. For adaptation to 2i, serum ESCs were transferred to 2i medium and cultured for >12 days (>6 passages) in 2i medium. ESCs used in this study include the female lines ES_Tsix-stop [39] and ES_Xist-del (a polymorphic 129:Cast female ESC line that shows non-random XCI due to a deletion in the Xist gene on the 129 allele [64]), and the male ESC lines E14Tg2a (E14) and Rex1GFPd2 lines [44, 65]). Derivation and culture of the EpiSCs was described previously [66, 67]. Derivation of NPC lines, including culture conditions and further details, has been described in Splinter et al. [35].

### EB differentiation of ESCs

Induction of ESC differentiation has been described by Barakat et al. [12]. In short, ESCs were split, and pre-plated on non-gelatinized cell culture dishes for 60 min. ESCs were then seeded in non-gelatinized bacterial culture dishes containing differentiation medium to induce EB formation. EB medium consisted of IMDM-glutamax, 15 % fetal calf serum, 100 U ml^−1^ penicillin, 100 mg ml^−1^ streptomycin, non-essential amino acids, 37.8 μl l^−1^ monothioglycerol and 50 μg ml^−1^ ascorbic acid. EBs were plated on coverslips 1 day prior to harvesting, and allowed to grow out.

### RNA isolation

Total RNA was isolated with Trizol (Invitrogen) according to the manufacturer’s recommendations. Total RNA (100 μg) was subjected to two rounds of poly(A) selection (Oligotex mRNA Mini Kit; QIAGEN), followed by DNaseI treatment (QIAGEN). mRNA (100–200 ng) was fragmented by hydrolysis (5× fragmentation buffer: 200 mM Tris acetate, pH8.2, 500 mM potassium acetate and 150 mM magnesium acetate) at 94 °C for 90 s and purified (RNAeasy Minelute Kit; QIAGEN). cDNA was synthesized using 5 μg random hexamers by Superscript III Reverse Transcriptase (Invitrogen). Double-stranded cDNA synthesis was performed in second strand buffer (Invitrogen) according to the manufacturer’s recommendations and purified (Minelute Reaction Cleanup Kit; QIAGEN). Strand-specific rRNA depleted double-stranded cDNA profiling used for the NPC lines was performed with the ScriptSeq kit (catalog number SS10924) from Illumina, according to the instructions of the manufacturer. rRNA depletion was performed with the Ribo-Zero rRNA Removal Kit using 5 μg of total RNA (Human/Mouse/Rat; catalog number RZH110424).

### Xist staining

RNA FISH analysis was performed as described previously [68, 69]. In short, differentiated ESCs were grown on coverslips, fixed in 4 % paraformaldehyde (PFA) in phosphate-buffered saline (PBS), and permeabilized with 0.2 % pepsin (4 min; 37 °C), followed by post-fixation using 4 % PFA/PBS at room temperature. The Xist probe was a cDNA sequence [53], which was digoxygenin labeled by nick translation (Roche). After overnight hybridization, slides were washed in 2× SSC (5 min; 37 °C), in 50 % formamide, 2× SSC (3 × 10 min; 37 °C), followed by washing in Tris-saline-tween. Target sequences were detected using fluorescently labeled antibodies detecting digoxygenin.

### Sequencing

For the poly(A)+ samples, cDNA was prepared for sequencing by end repair of 20 ng double-stranded cDNA as measured by Qubit (Invitrogen). Adaptors were ligated to DNA fragments, followed by size selection (~300 bp) and 14 cycles of PCR amplification. Quality control of the adaptor-containing DNA libraries of both poly(A)+ and ScriptSeq samples was performed by quantitative PCR and by running the products on a Bioanalyzer (BioRad). Cluster generation and sequencing (32–42 bp) was performed with the Illumina Genome Analyzer IIx or Hi-Seq 2000 platforms according to standard Illumina protocols. Generation of FASTQ files and demultiplexing was performed using Illumina CASAVA. All sequencing analyses were conducted based on the *M. musculus* NCBI m37 genome assembly (MM9; assembly July 2007). Additional file 2: Table S1 and Additional file 3: Table S2 summarize the sequencing output. All RNA-seq data (FASTQ, BED, and WIG files), as well as the allelic counts over individual polymorphic sites for each of the Tsix-stop profiles, are present in the NCBI Gene Expression Omnibus (GEO) SuperSeries GSE60738.

### Polymorphic sites between the genomes of the 129 and Cast mouse species

Known polymorphic sites between the mouse species 129 and Cast (nucleotide substitutions, not indels) were collected using polymorphic sites determined by (i) the Sanger mouse sequencing project using the March 2011 release [70, 71] (we used [72] for the species 129S1, C57BL and CAST) and (ii) the NIEHS/Perlegen mouse resequencing project [73] (we used the b04_Chr*_genotype.dat files for the species 129S1/SvImJ and CAST/EiJ [74] and the C57BL/6 J reference genome of NCBI Build 36 [75]). This resulted in a total of 20,785,351 polymorphic sites between the genomes of 129 and Cast.

### Allele-specific mapping using GSNAP

FASTQ files were mapped using GSNAP version 2011-03-10 [46]. To avoid bias in the mapping of either the Cast- or the 129-derived reads, the alternative alleles of polymorphic sites between the 129 and Cast genome (see above) are included in the reference during mapping (GSNAP SNP-tolerant mapping; flag –v). Only sequence tags aligning to a single position on the genome were considered for further analysis on the 32–42 bp aligned sequence reads. The output data were converted to Browser Extensible Data (BED) files for quantification, Wiggle (WIG) files for viewing and GSNAP output files for determining allelic bias per gene. To obtain RNA-seq gene expression values (RPKM), we used Genomatix [76] (ElDorado 12–2010) selecting RefSeq genes (NCBI m37 genome assembly; MM9; Additional file 3: Table S2).

### Calling of allele-specific gene expression

Within the individual samples, we used the mapped tags to determine the sequence tag coverage per allele for each of the 20,785,351 polymorphic sites using GSNAP tally. A total of 4,888,065 polymorphic sites were covered at least once in any of the samples used for this study. Per single polymorphic nucleotide, the pile-ups were subsequently assigned to either the 129 or the Cast allele using custom Perl-based scripts (the allelic counts over individual polymorphic sites for each of the Tsix-stop profiles are present within GEO GSE60738). To avoid including counts from positions which were reported to be polymorphic in the Sanger sequencing project and/or the NIEHS/Perlegen resequencing projects, but which were not present in the genotypes used for the current study, we selected polymorphic sites that were covered at least twice from both the 129 and the Cast allele. This resulted in a total of 1,121,809 polymorphic sites used in further analysis. Counts over polymorphic sites within exons of individual RefSeq genes for either 129 or Cast were summed to obtain allele-specific gene expression counts for both species (Additional file 3: Table S2). The ratio between the 129 counts or the Cast counts versus the total counts (129 + Cast) represent the relative contribution of the 129 or Cast allele, respectively, to expression of a particular gene. To calculate absolute allele-specific expression values, we multiplied the relative contribution of either 129 or Cast with the total RPKM expression value of a gene. For the ESC differentiation time course, only genes that contained a count of >80 over the complete time course from both the 129 as well as from the Cast allele were included for further analysis as further explained in the main text and in Additional file 1: Figure S4b.

### Consistency of allelic bias per polymorphic site over the full transcript

Genes containing a single polymorphic site and fulfilling the criteria as described above were included in the analysis for the EB differentiation time course. In case multiple polymorphic sites were included in the allele-specific gene expression calling for a given gene (see previous section), we evaluated the consistency in allelic ratio over the individual polymorphic sites. For genes containing at least two polymorphic sites showing a coverage of more than nine counts over the ESC differentiation time course from either the 129 or Cast allele, the relative contributions from 129 and Cast were calculated for these individual sites. Genes that showed a standard error of the mean (STDEM) of >0.1 over the individual polymorphic sites were excluded from further analysis.

### Escape from XCI in NPC lines

Genes were considered escape genes if they fulfilled the following criteria: (i) at least two polymorphic sites showing signals from the Xi; (ii) more than two counts originating from the Xi; (iii) a relative contribution of >10 % from the Xi to the total gene expression (similar to Yang et al. [37]; Table 1; Additional file 5: Table S4).

### Clustering, GSEA and statistical testing of distributions (boxplots)

For clustering, changes of Xi/Xa ratios (in log2) relative to undifferentiated ESCs (T = 0) were calculated over the differentiation time course. K-means clustering was performed using the TIGR Multi experiment viewer (TMEV) version 4.0. GSEA [77] was performed using Gene Trail [78]. The rank of the individual genes in each cluster among all 259 genes was determined based on the distance of each gene to the XIC. Statistical testing on distributions represented by boxplots was performed according to McGill et al. [47] by comparing the notches of the boxplots. The notches extend 1.58× Interquartile range/Square root(n) and give an accurate estimate of the 95 interval for comparing medians, whereby boxplots with non-overlapping notches are significantly different (*p* < 0.05 [47]).

### Imprinting in undifferentiated ESCs

Genes were considered imprinted according to the following two criteria: (i) for at least two polymorphic sites, at least 80 % of these sites show the same allelic bias towards either 129 or Cast with binomial *p* < 0.01; (ii) the total relative contribution of either 129 or Cast to the total expression of a gene is >75 %.

### Sanger sequencing of cDNA

cDNA was synthesized from 2 μg total RNA using 1 μg random hexamers by Superscript III Reverse Transcriptase (Invitrogen). PCR fragments for sequencing were obtained using the Phusion High Fidelity DNA Polymerase kit (NEB M0530C) on the synthesized cDNA, followed by purification of the PCR products using Agencourt AMPure (Beckman Coulter). Further details on the conditions of the PCR, as well as on the PCR primers and sequencing primers used, are listed in Additional file 7: Table S6. Sanger sequencing was performed on the 3730 Sequence Analyzer (Life Technologies) using Big Dye Terminator version 1.1 according to standard protocols.

### Hi-C (data) analysis

Collection of the cells for Hi-C and the Hi-C sample preparation procedure was performed as previously described [79], with the slight modification that DpnII was used as restriction enzyme during initial digestion. Paired-end libraries were prepared according to Lieberman-Aiden et al. [79] and sequenced on the NextSeq 500 platform using 2 × 75 bp sequencing (Additional file 2: Table S1). Reads were mapped to the reference mouse genome (mm9) using BWA MEM [80] with default parameters. Reads were filtered based on mapping quality score (mapQ ≥10) and PCR duplicates were removed. (Normalized) interaction matrices at a resolution of 40 kb and the corresponding two-dimensional heat maps were generated as previously described [79] using optimized LGF normalization (normLGF) from the R package HiTC [81]. The Hi-C Domain Caller package was used to calculate the directionality index from the normalized interaction matrix and to determine domains and boundaries using default parameters [51]. For allele-specific domain calling, we first filtered the 129-derived sequence tags using intersection and assignment based on the polymorphic sites between Cast and 129 (20,785,351 polymorphic sites as reported above). Allele-specific domain calling was identical to the procedure for the total data set.

### Correlation between Hi-C experiments and Hi-C TAD boundaries

Correlations and overlaps for the Hi-C experiments were calculated according to Dixon et al. [51]. In short, the correlation between Hi-C experiments was calculated by the Spearman’s rank correlation coefficient for each 40 kb bin based on the number of interactions (signal) within the 25 bins upstream and 25 bins downstream. For overlap of boundaries, we considered a cutoff of ≤40 kb of boundaries between samples. The Spearman’s rank correlation coefficient for TAD boundaries was based on ten 40-kb bins upstream and downstream of the boundaries of two samples. For random correlation, we 2000 times randomly selected 20 bins from each of the two experiments and calculated correlations.

### Other datasets used

RNA-seq of the ESC line E14 (male; 2i and serum) was obtained from Marks et al. [44]. RNA-seq of EpiSC lines was obtained from Veillard et al. [67] and includes one newly generated profile from a female EpiSC that was obtained by nuclear transfer (NT) [66]. Three-dimensional genome organization by Hi-C of male serum J1 ESCs grown on feeders was obtained from Dixon et al. [51]. For human, the Hi-C was generated from the female fibroblast line IMR90 [51]. Additional data of escape genes in NPCs was obtained from Gendrel et al. [52]. Escape genes from human were obtained from Carrel and Willard [29], and were profiled in hybrid lines generated from human female fibroblasts and mouse cells. Genes were considered as escapers in case of a ratio >5/9 in the hybrid lines.

## Additional files

Additional file 1: Figure S1. Decreased *Xist* expression and increased expression of X‐linked genes in ES_Tsix‐stop ESCs maintained in serum‐free media in the presence of 2 kinase inhibitors (“2i”) compared with ES_Tsix‐stop ESCs maintained under serum condiPons. **Figure S2.** Validation of the differentiation times course of the female ES_Tsix‐stop ESCs towards Embryoid Bodies (EBs) by RNA expression of marker genes. During EB formation, expression of the pluripotency markers decreased five‐ to tenfold, while markers for the three germlayers are induced. **Figure S3.** Applying RNA‐seq to detect genomic abnormalities in ES_Tsix‐stop ESCs. **Figure S4.** Characteristics of allele‐specific mapping. **Figure S5.** Dynamics of XCI for genes having high (RPKM >2) or low (RPKM ≤2) mean expression over the time course, showing that lowly expressed genes show faster XCI dynamics compared with highly expressed genes. **Figure S6.** Genome browser views (not allele‐specific) of the genes as shown in Fig. 4b. **Figure S7.** Expression dynamics of genes within the four clusters as characterized in Fig. 4. At all time points, genes within cluster 3 (the “late” cluster) are significantly higher expressed compared with genes in the other clusters. **Figure S8.** Overlap of the genes within the clusters identified in this study (on the *x‐axis*) with the genes within the clusters identified by Lin et al. [23] (*colored*), showing poor overlap between both studies for corresponding clusters. **Figure S9.** Running‐sum statistics for the genes present within each of the four clusters versus all 259 genes included in the analysis based on the distance to the XIC. **Figure S10** Spread of gene silencing during XCI over the Xi. The trend line (polynomial order 3) of the Xi/Xa raPo per gene over the X chromosome is plotted per time point after the onset of EB differentiation. **Figure S11.** X‐linked genes with allelic bias in undifferentiated female 2i ESCs are randomly distributed over the X chromosome. **Figure S12.** Genes within region 1 are significantly higher expressed in the NPCs in which they escape XCI compared with the NPCs in which they are silenced on the Xi. **Figure S13.** ValidaPon of genes escaping XCI in the three escape regions using cDNA Sanger sequencing. **Figure S14.** The three escape regions are stably maintained during propagation of NPCs. **Figure S15.** Distribution of ES_Tsix‐stop Hi‐C sequence tags over the genome shows that the ES_Tsix‐ stop ESCs contains no major genomic abnormalities besides trisomy 12. **Figure S16.** Domains and boundaries on autosomes as well as on chromosome X are largely stable between female ES_Tsix‐stop and male J1 ESCs [51]. **Figure S17.** The three regions escaping XCI colocalize with TADs as identified in ES_Tsix‐stop ESCs. **Figure S18.** Validation of the three regions escaping XCI and their associaPon with TADs. **Figure S19.** In human, clusters of genes escaping XCI on the short arm of chromosome X [29] colocalize with topologically associated domains (TADs) [51]. (PDF 204 kb) Additional file 2: Table S1. RNA-seq and Hi-C profiles generated for this study. (XLSX 11 kb) Additional file 3: Table S2. Genome-wide gene expression values and allelic counts for the profiles generated from the ES_Tsix-stop cells (*tab 1*, undifferentiated ESCs, EB time course and NPCs) and the ES_Xist-del cells (*tab 2*, undifferentiated ESCs). (XLSX 8327 kb) Additional file 4: Table S3. Detailed overview of the overlap between genes in the clusters identified in this study and genes in similar clusters as identified by Lin et al. [23]. (XLSX 61 kb) Additional file 5: Table S4. Imprinting status of all X-linked genes in NPCs. (XLSX 421 kb) Additional file 6: Table S5. Coordinates of TADs as identified in the female ES_Tsix-stop ESCs. (XLSX 95 kb) Additional file 7: Table S6. Primers used for PCR on cDNA and for Sanger sequencing. (XLSX 47 kb)

## Abbreviations

129: *M.m. musculus* 129/SV-Jae
bp: base pair
Cast: *M.m. castaneus*
E: embryonic day
EB: embryoid body
EpiSC: epiblast stem cell
ESC: embryonic stem cell
FISH: fluorescent in situ hybridization
GEO: Gene Expression Omnibus
GSEA: gene set enrichment analysis
GSNAP: Genomic Short-read Nucleotide Alignment Program
H3K27me3: trimethylated lysine 27 on histone 3
*M.m.*: *Mus musculus*
MAPK: mitogen-activated protein kinase
NPC: neural progenitor cell
PBS: phosphate-buffered saline
PFA: paraformaldehyde
PRC: Polycomb repressive complex
SNP: single-nucleotide polymorphism
TAD: topologically associating domain
Xa: active X chromosome
XCI: X chromosome inactivation
Xi: inactivate X chromosome
XIC: X-inactivation center
