# Erratum to: Dynamics of gene silencing during X inactivation using allele-specific RNA-seq

**DOI:** 10.1186/s13059-016-0885-4

**Published:** 2016-02-05

**Authors:** Hendrik Marks, Hindrik H. D. Kerstens, Tahsin Stefan Barakat, Erik Splinter, René A. M. Dirks, Guido van Mierlo, Onkar Joshi, Shuang-Yin Wang, Tomas Babak, Cornelis A. Albers, Tüzer Kalkan, Austin Smith, Alice Jouneau, Wouter de Laat, Joost Gribnau, Hendrik G. Stunnenberg

**Affiliations:** 1 Radboud University, Faculty of Science, Department of Molecular Biology, Radboud Institute for Molecular Life Sciences (RIMLS), Nijmegen, 6500HB The Netherlands; 2 Radboud University, Faculty of Science, Department of Molecular Developmental Biology, Radboud Institute for Molecular Life Sciences (RIMLS), Nijmegen, 6500HB The Netherlands; 3 Department of Reproduction and Development, Erasmus MC, University Medical Center, Rotterdam, The Netherlands; 4 Hubrecht Institute, University Medical Center Utrecht, Uppsalalaan 8, Utrecht, 3584CT The Netherlands; 5 Biology Department, Queen’s University, Kingston, ON Canada; 6 Wellcome Trust-Medical Research Council Stem Cell Institute, University of Cambridge, Tennis Court Road, Cambridge, CB2 1QR UK; 7 INRA, UMR1198 Biologie du Développement et Reproduction, Jouy-en-Josas, F-78350 France

After the publication of this work [1], we noticed there was an error in Fig. 5 where −1,0 and 1 are incorrectly displayed in the y-axis in panel b. Please see the corrected Fig. 5 below. We apologize for this error.

**Fig. 5:**
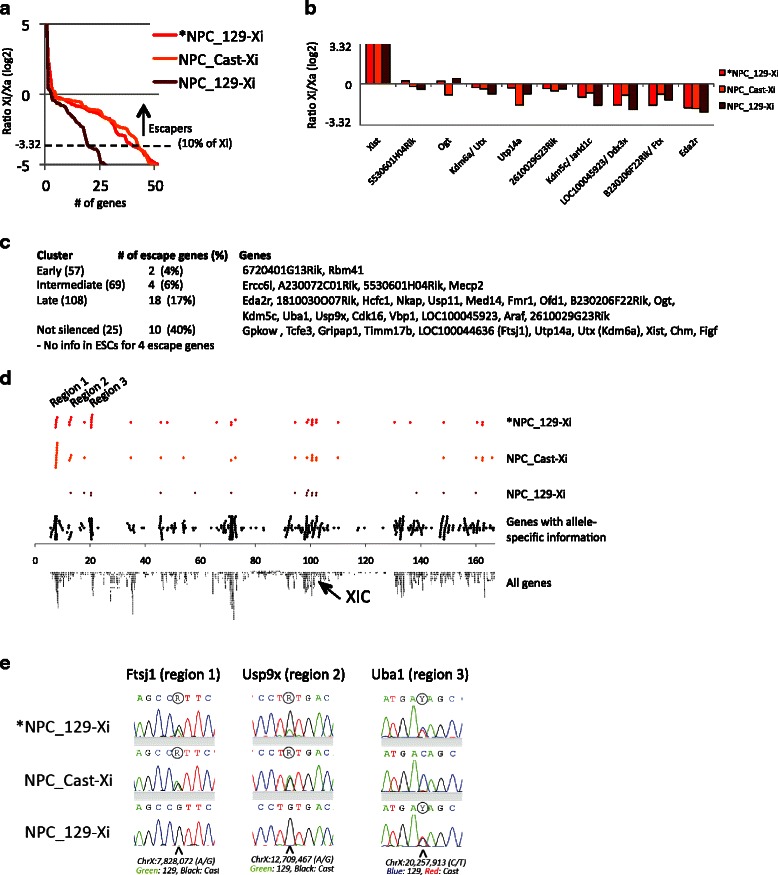
llele-specific RNA-seq on three NPC lines identifies three distal regions of genes that escape XCI. **a** Ratio of Xi/Xa (*y-axis*; for each of the three NPC lines sorted from highest to lowest) for genes showing a log2 ratio of at least −5. We set the cutoff for escape on 10 % relative expression from the Xi versus the Xa (log 2 ratio of > −3.32; similar to Yang et al. [37]). **b** Xi/Xa ratio of genes that escape XCI in all three NPC lines. **c** Distribution of the escape genes identified in *NPC_129-Xi over the four clusters as characterized in Fig. 4a. **d** Localization of the escape genes within each NPC line over the linear X chromosome (see also Table 1). The *black dots* on the *fourth row* represent all X-linked genes for which high-confidence allele-specific ratios were obtained in NPCs. **e** Validation of the escape genes within the three escape regions by Sanger sequencing of cDNA. See Additional file 1: Figure S13 for the full panel of 13 genes that we validated, and for further details
