# Supplementary material for: Dynamics of gene silencing during X inactivation using allele-specific RNA-seq
Source: Genome Biol. 2015 Aug 3;16(1):149. doi: 10.1186/s13059-015-0698-x (PMC4546214; doi:10.1186/s13059-015-0698-x)
Supplement: Additional file 1: Figure S1. — Decreased Xist expression and increased expression of X‐linked genes in ES_Tsix‐stop ESCs maintained in serum‐free media in the presence of 2 kinase inhibitors (“2i”) compared with ES_Tsix‐stop ESCs maintained under serum condiPons. Figure S2. Validation of the differentiation times course of the female ES_Tsix‐stop ESCs towards Embryoid Bodies (EBs) by RNA expression of marker genes. During EB formation, expression of the pluripotency markers decreased five‐ to tenfold, while markers for the three germlayers are induced. Figure S3. Applying RNA‐seq to detect genomic abnormalities in ES_Tsix‐stop ESCs. Figure S4. Characteristics of allele‐specific mapping. Figure S5. Dynamics of XCI for genes having high (RPKM >2) or low (RPKM ≤2) mean expression over the time course, showing that lowly expressed genes show faster XCI dynamics compared with highly expressed genes. Figure S6. Genome browser views (not allele‐specific) of the genes as shown in Fig. 4b. Figure S7. Expression dynamics of genes within the four clusters as characterized in Fig. 4. At all time points, genes within cluster 3 (the “late” cluster) are significantly higher expressed compared with genes in the other clusters. Figure S8. Overlap of the genes within the clusters identified in this study (on the x‐axis) with the genes within the clusters identified by Lin et al. [23] (colored), showing poor overlap between both studies for corresponding clusters. Figure S9. Running‐sum statistics for the genes present within each of the four clusters versus all 259 genes included in the analysis based on the distance to the XIC. Figure S10 Spread of gene silencing during XCI over the Xi. The trend line (polynomial order 3) of the Xi/Xa raPo per gene over the X chromosome is plotted per time point after the onset of EB differentiation. Figure S11. X‐linked genes with allelic bias in undifferentiated female 2i ESCs are randomly distributed over the X chromosome. Figure S12. Genes within region 1 are significantly hi [file 13059_2015_698_MOESM1_ESM.pdf]

Additional File 1 (Supplementary figures) belonging to Marks et al. (2015), Genome Biology: Dynamics of gene silencing during X inactivation using allele-specific RNA-seq

Suppl Figure 1

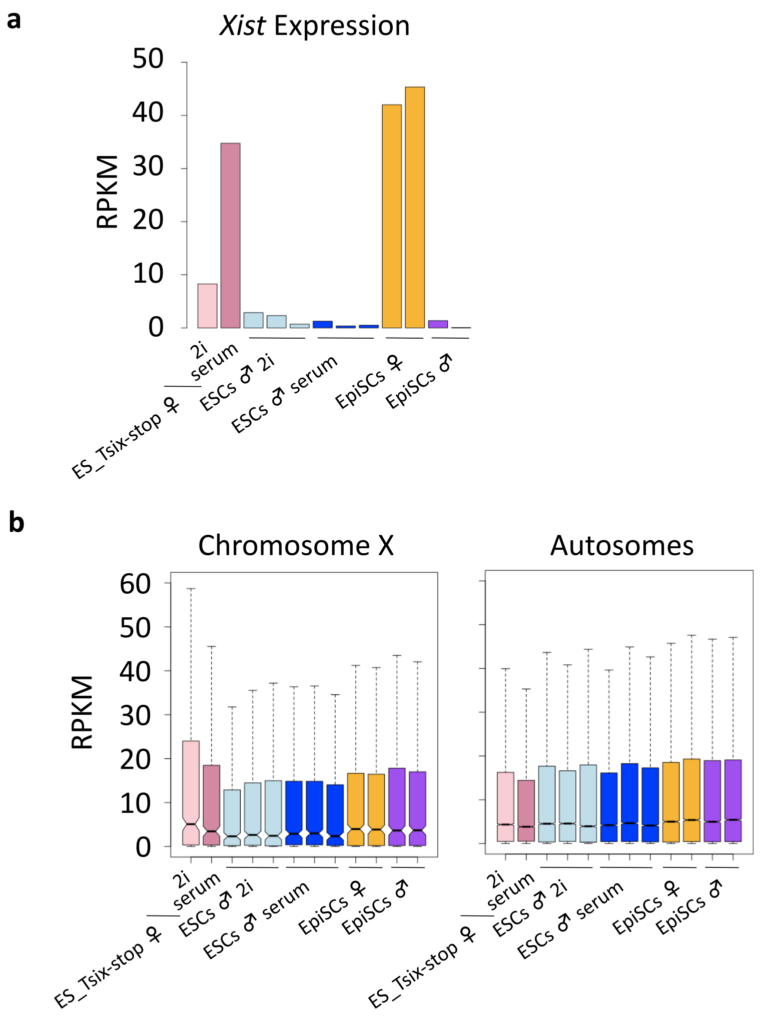

Supplementary Figure 1. Decreased *Xist* expression and increased expression of X-linked genes in ES_Tsix-stop ESCs maintained in serum-free media in the presence of 2 kinase inhibitors ("2i") compared with ES_Tsix-stop ESCs maintained under serum conditions.

(a) *Xist* gene expression in female 2i or serum ES_Tsix-stop ESCs. For comparative purposes, we included male 2i or serum ESCs and epiblast stem cells (*EpiSCs*) in this analysis. Male ESCs are included as a control for gene expression in the presence of a single active X chromosome. Female EpiSCs have undergone random XCI and stably maintain their Xi [82]. Male 2i ESCs include two 2i-adapted E14 ESC lines [44], as well as one 2i-adapted Rex-GFP ESC line [65]; male serum ESCs include E14 and two Rex-GFP ESC lines, all derived and maintained in serum-containing medium [44, 65]. Derivation of male and female pluripotent EpiSCs from post-implantation epiblast cells (E6.5) has been described previously [66, 83, 84]. See "Materials and methods" for more information about these cell lines.

(b) Distribution of gene expression in the corresponding cell lines as shown in (**a**). To be included in this analysis, genes were required to show expression levels of RPKM > 0.5 in any of the cell lines (637 and 16073 genes on the X chromosome and on autosomes, respectively). In line with the presence of two active X chromosomes, female Tsix-stop ESCs (2i and serum) show higher expression of X-linked genes relative to male ESCs (2i and serum) ($p < 0.05$ for 2i female versus male ESCs [47]). Gene expression between male and female EpiSC is similar as both only contain a single active X chromosome.

Suppl Figure 2

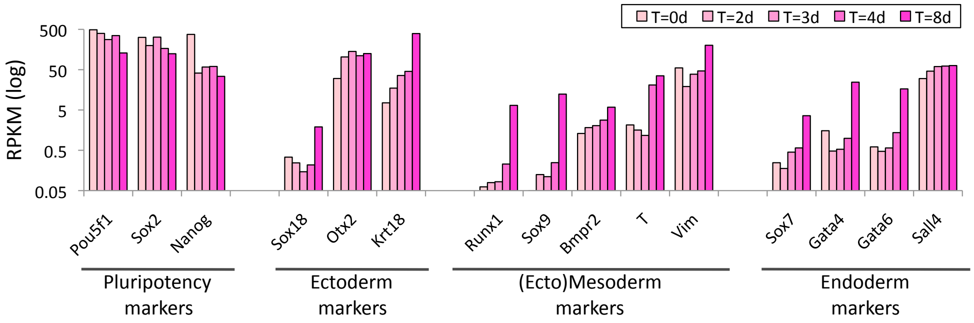

Supplementary Figure 2. Validation of the differentiation time course of the female ES_Tsix-stop ESCs towards Embryoid Bodies (EBs) by RNA expression of marker genes. During EB formation, expression of the pluripotency markers decreased five- to tenfold, while markers for the three germlayers are induced.

Suppl Figure 3

a

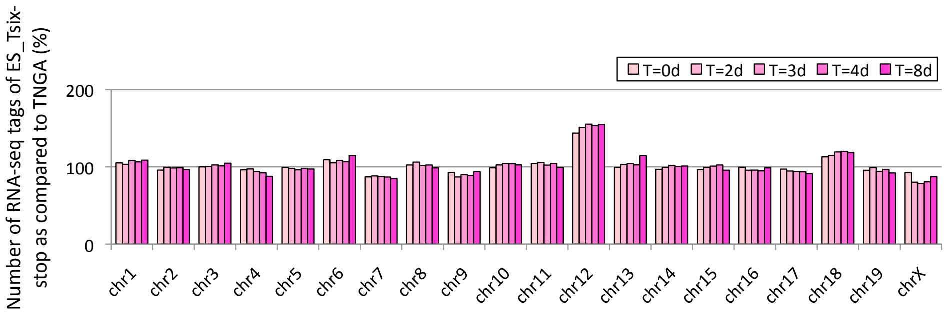

b

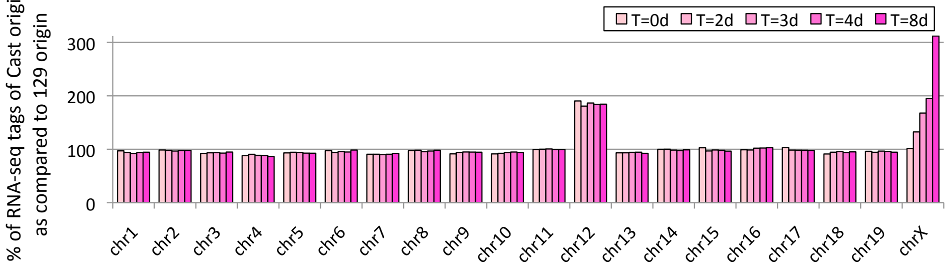

Supplementary Figure 3. Applying RNA-seq to detect genomic abnormalities in ES_Tsix-stop ESCs.
(a) Distribution of all RNA-seq sequence tags of the female ES_Tsix-stop ESCs over chromosomes compared with the karyotypic-normal undifferentiated female TNGA 2i ESCs [44]. TNGA ESCs are a mouse ES cell line with a green fluorescent protein (GFP) insertion into one of the Nanog loci. The 50% higher number of tags present on chromosome 12 suggests a trisomy 12 in the ES_Tsix-stop, which has been observed before in other ESC lines [85]. This is in line with the observations made by Hi-C as reported in Fig. S15. Besides chromosome 12, the distribution of tags over the chromosomes is very similar between TNGA and ES_Tsix-stop. This suggests that the ES_Tsix-stop ESCs contain no major genomic abnormalities besides trisomy 12. **(b)** Allele-specific distribution of RNA-seq sequence tags of ES_Tsix-stop ESCs over all chromosomes, plotted as Cast/129 (%). The double number of sequence tags from Cast on chromosome 12 suggests that the trisomy 12 observed in the ES_Tsix-stop ESCs is caused by two copies of chromosome 12 of Cast origin, next to the chromosome 12 of 129 origin present in these ESCs. The dynamics observed on chromosome X represent XCI during which the 129-derived chromosome is inactivated.

Suppl Figure 4

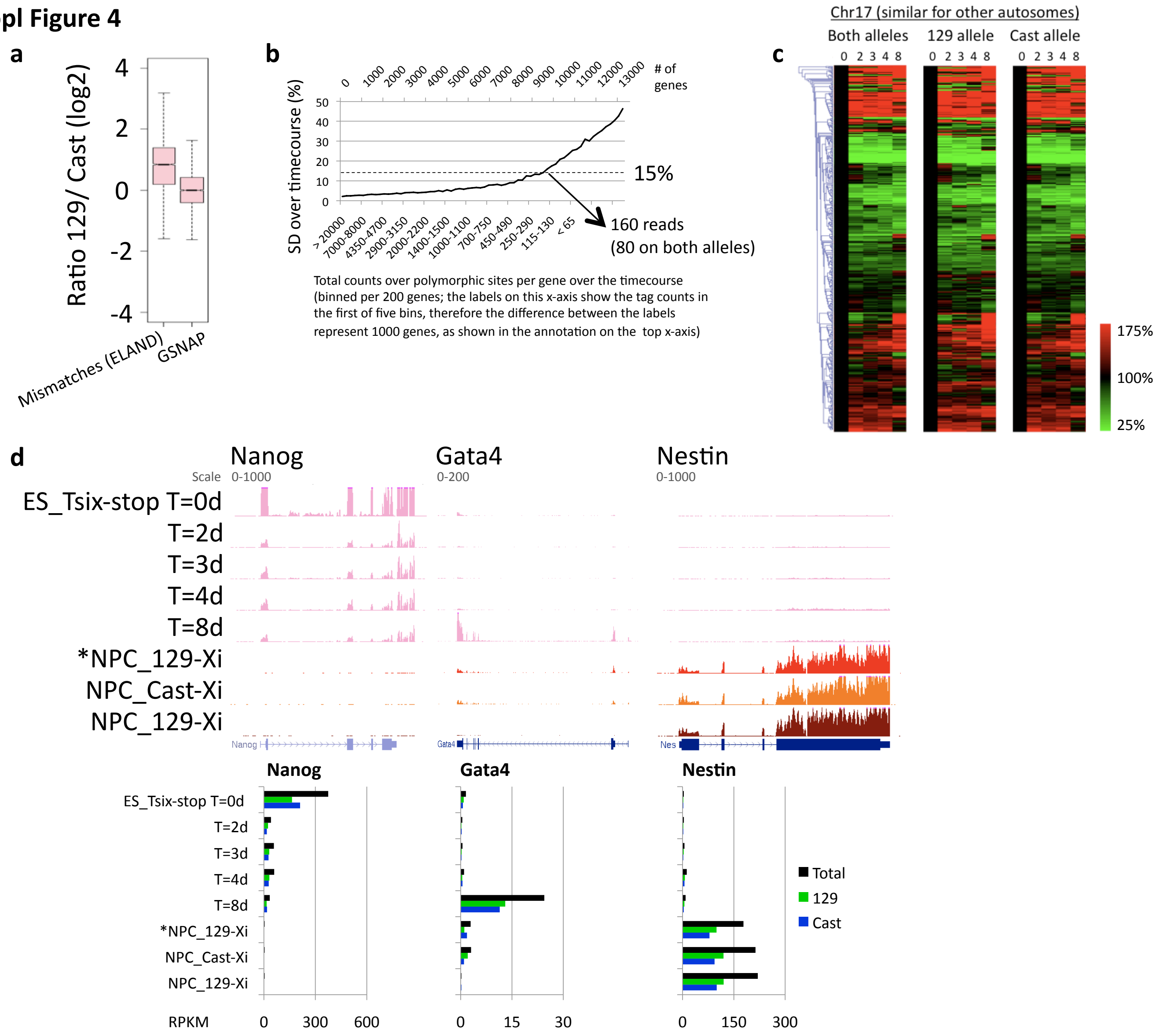

Supplementary Figure 4. Characteristics of allele-specific mapping.

(a) Distribution of relative gene expression from the 129- versus the Cast-derived allele in undifferentiated ES_Tsix-stop ESCs using two different allele-specific pipelines. A log2 ratio of 0 represents biallelic equal gene expression from the 129 and Cast alleles, while positive and negative ratios represent higher expression from the 129 or Cast allele, respectively. The ELAND-based pipeline maps against the B6 mouse reference genome and uses mismatches to determine 129- and Cast-derived reads (for example Marks et al. [22]). Since the nucleotide composition of the B6 reference genome is much closer to the 129 genome than to the Cast genome, this results in preferential mapping of 129-derived reads and therefore a considerable bias towards expression from the 129-derived genome. The GSNAP-based pipeline includes the alternative alleles of polymorphic sites between the 129- and Cast-genome during mapping. This results in an unbiased assignment of reads with equal contribution of reads derived from 129 and Cast, respectively. **(b)** Standard deviation (SD) of allelic expression for autosomal genes (for which the allelic ratio is expected to be stable) over the five individual time points, as a function of the total counts over the polymorphic sites over the time course. Per time point, the relative contribution of 129 and Cast to the expression of each autosomal gene (in percent) was calculated, after which the SD over the 5 time points was determined for each gene. This analysis shows that lower coverage of genes result in higher SDs and thus in less accurate measurements of allelic ratios. For the current time course, we included genes that showed a SD of <15 % corresponding to a total count of 160 (at least 80 from each allele) of reads over the polymorphic sites of a gene. **(c)** Heatmap for total gene expression on chromosome 17. For all genes with allele-specific information, we plotted the changes in total gene expression (*left*), as well as the allele-specific changes (*middle* and *right panel*) during EB formation (T=0 undifferentiated ES_Tsix-stop ESCs set at 100%). Since the middle and right heatmap are nearly identical to the left heatmap, most changes occur equally from both alleles, as expected for autosomes. **(d)** Representative examples: expression dynamics of three marker genes (Nanog: pluripotency; Gata4: primitive endoderm; Nestin: neural). The *bottom panel* shows total expression levels in RPKM corresponding to the genome browser views, as well the relative expression from 129 and Cast. Changes in expression for these genes occur equally from both alleles.

Suppl Figure 5

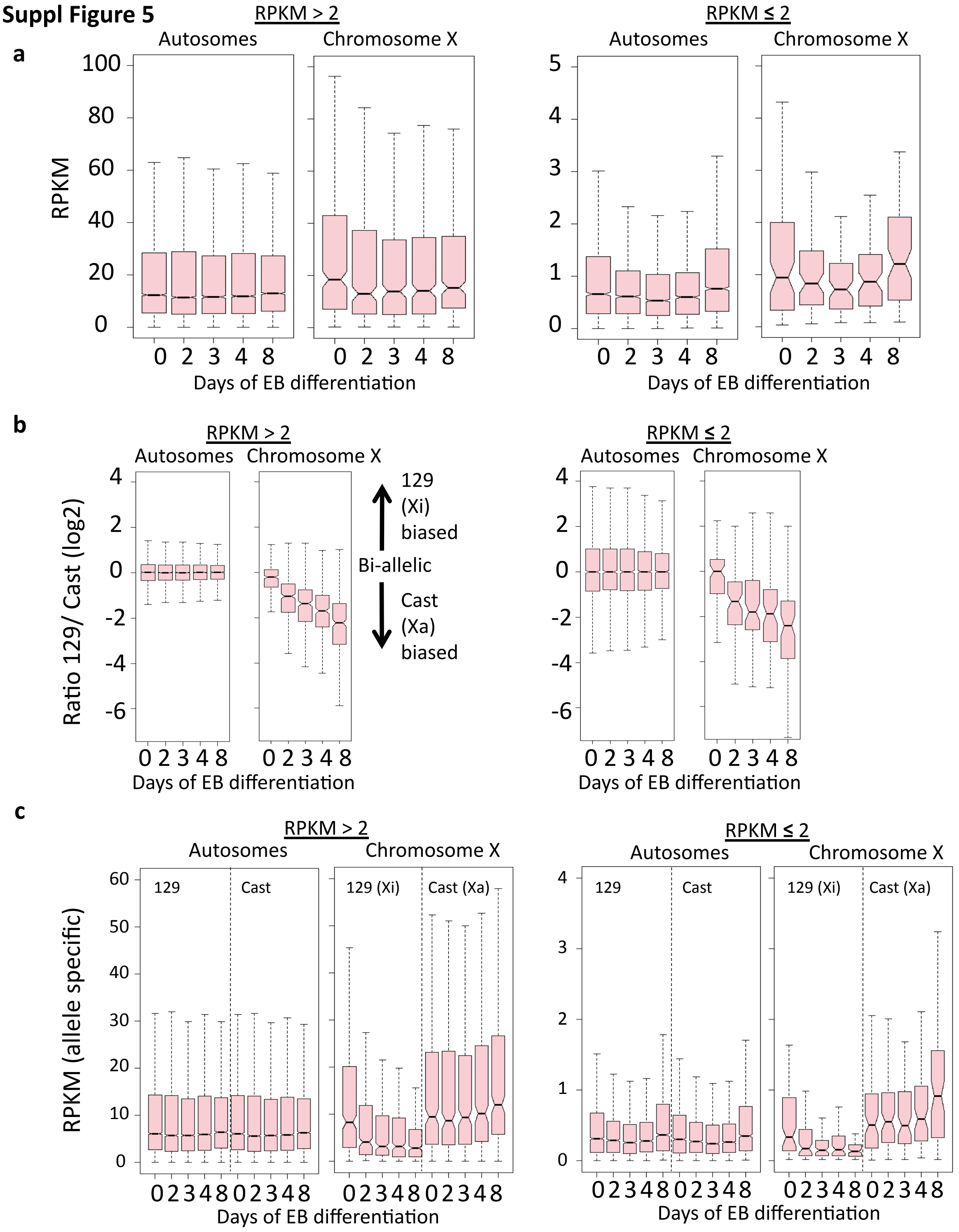

Supplementary Figure 5. Dynamics of XCI for genes having high (RPKM >2) or low (RPKM ≤2) mean expression over the time course, showing that lowly expressed genes show faster XCI dynamics compared with highly expressed genes. Fig. 3c shows a direct comparison of the median ratios for both categories over the time course as is also visible in (**b**) (by comparing both X chromosome panels). For this analysis, we used milder criteria to facilitate analysis of lowly expressed genes: all genes showing RPKM > 0.05 in at least one of the time points, and for which we obtained an allelic ratio for all time points, were included [338/ 81 X-linked genes and 8612/ 2528 autosomal genes (highly/ lowly expressed), respectively].

(a) Distribution of gene expression in female (ES_Tsix-stop) ESCs during EB formation for autosomal genes and X-linked genes as in Fig. 2c. The left plot shows genes with an average expression level of RPKM >2 over the time course. The right plot shows genes with an average expression level of RPKM ≤2 over the time course. **(b)** Distribution of relative expression of genes from the 129 versus the Cast allele during EB formation of ES_Tsix-stop as in Fig. 3a, separated for highly expressed genes (*left*) and lowly expressed genes (*right*). The ratios for T = 2d, T = 3d and T = 4d are significantly lower for the lowly expressed genes compared with the highly expressed genes for the X chromosome (as visible from the non-overlapping notches of the *left panel* for chromosome X (RPKM >2) in comparison to the *right panel* for chromosome X (RPKM ≤2) for these timepoints [47]), indicating that lowly expressed genes show significantly faster XCI dynamics. **(c)** Distribution of absolute gene expression from the 129 and Cast alleles (allelic expression values in RPKM) in the ES_Tsix-stop ESCs during EB formation for highly and lowly expressed genes, as in Fig. 3b.

Suppl Figure 6

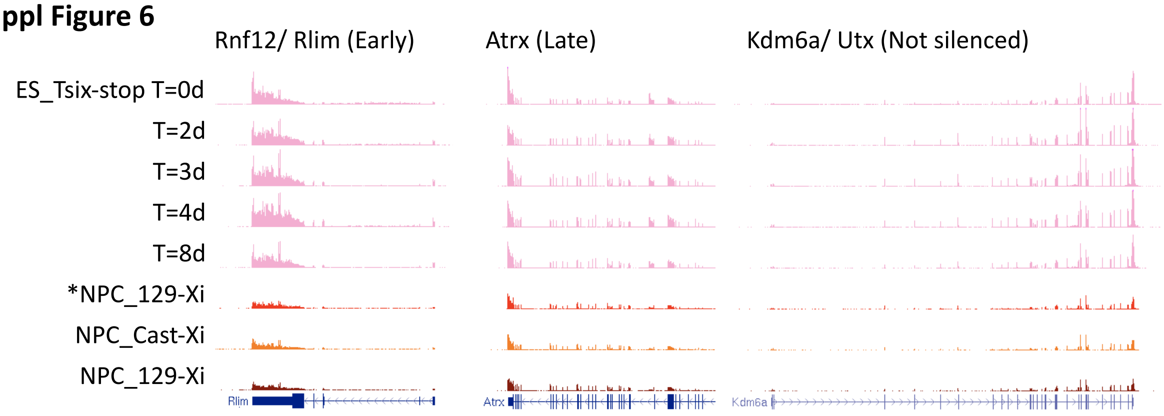

Supplementary Figure 6. Genome browser views (not allele-specific) of the genes as shown in Fig. 4b.

Suppl Figure 7

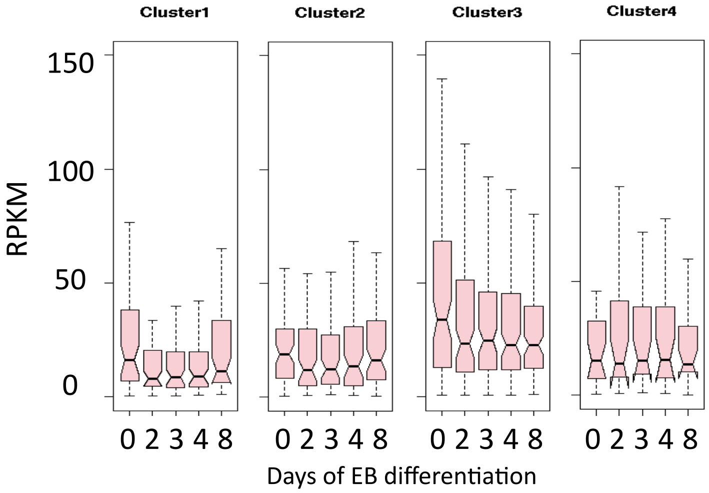

Supplementary Figure 7. Expression dynamics of genes within the four clusters as characterized in Fig. 4. At all time points, genes within cluster 3 (the "late" cluster) are significantly higher expressed compared with genes in the other clusters.

Suppl Figure 8

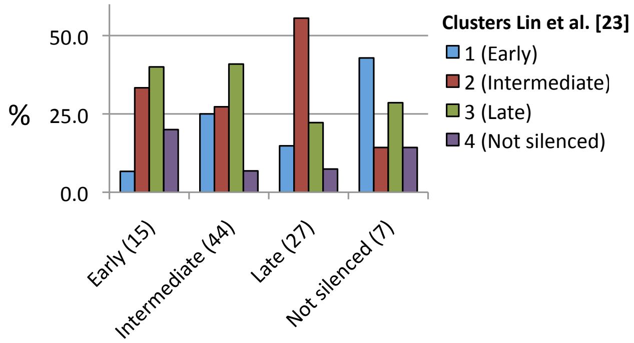

Supplementary Figure 8. Overlap of the genes within the clusters identified in this study (on the *x-axis*) with the genes within the clusters identified by Lin et al. [23] (*colored*), showing poor overlap between both studies for corresponding clusters. Per cluster identified in this study, the percentage of overlap with the genes present in the clusters identified by Lin et al. [23] (*y-axis*) is shown. There were 93 genes assayed by both studies and therefore included in this analysis. Table S3 contains a detailed overview of the overlap.

Suppl Figure 9

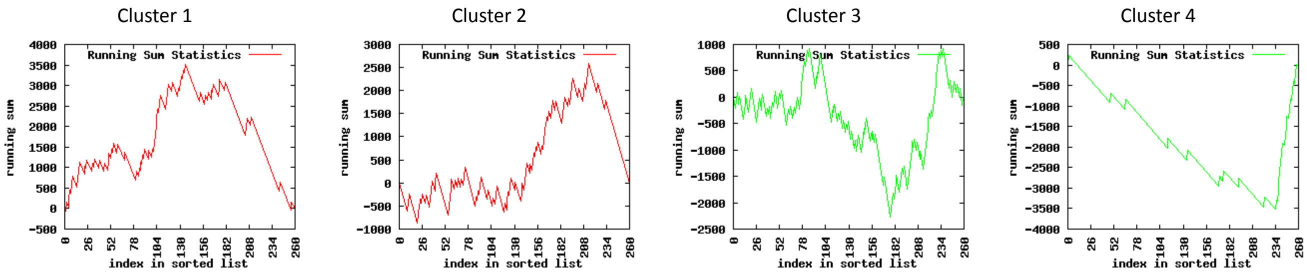

Supplementary Figure 9. Running-sum statistics for the genes present within each of the four clusters versus all 259 genes included in the analysis based on the distance to the XIC. We ranked the genes according to their distance from the XIC. Then the entire ranked list is used to assess how the genes of each of the four clusters are distributed across the ranked list. To do this, GSEA walks down the ranked list of genes, increasing a running-sum statistic when a gene belongs to the set and decreasing it when the gene does not. *P* values of this analysis are documented in Fig. 4c (*p* value GSEA rank test) [74].

Suppl Figure 10

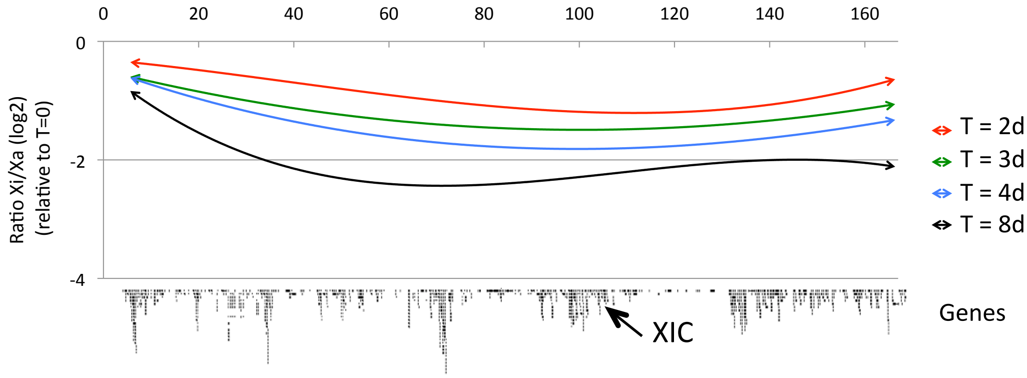

Supplementary Figure 10. Spread of gene silencing during XCI over the Xi. The trend line (polynomial order 3) of the Xi/Xa ratio per gene over the X chromosome is plotted per time point after the onset of EB differentiation. See Fig. 4d for more information (the *red T = 2d* trend line is the same as plotted in Fig. 4d).

Suppl Figure 11

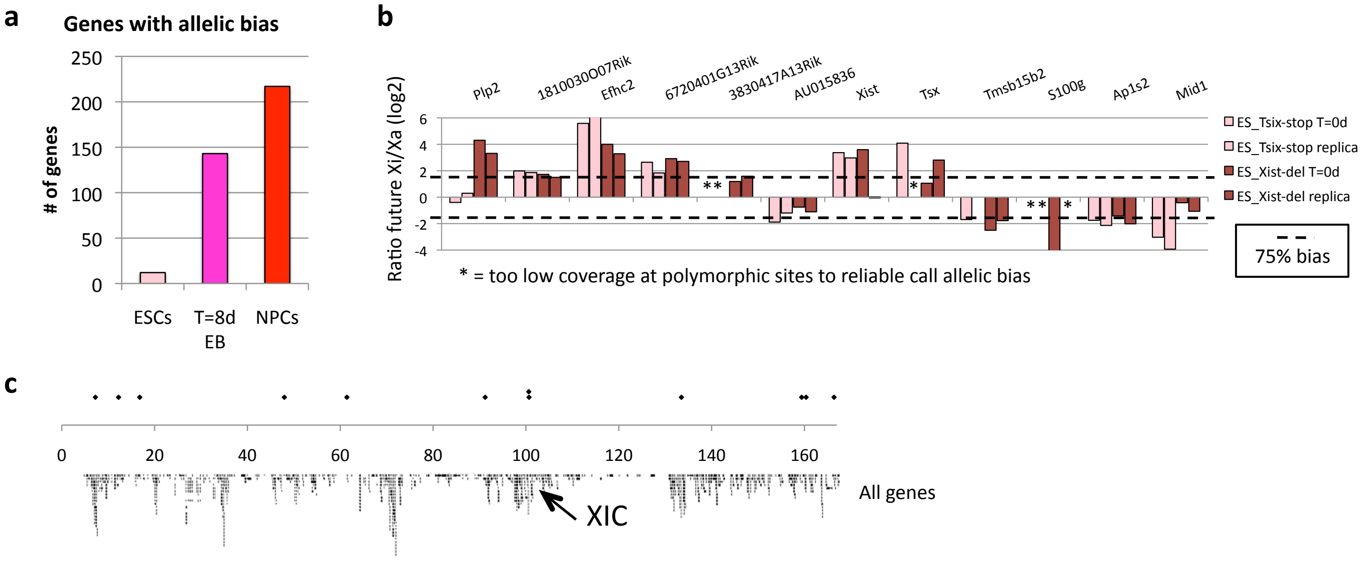

Supplementary Figure 11. X-linked genes with allelic bias in undifferentiated female 2i ESCs are randomly distributed over the X chromosome.

(a) Validation of the method for identification of genes showing allelic preference. To validate our method, we determined the number of X-linked genes showing allelic preference in any of the ESCs (*pink*), after 8-day EB formation (*purple*), or in any of the NPCs (*red*) (Table S4) using criteria as described in the "Materials and methods". The pipeline robustly detects biased (XCI) genes after 8-day EB formation and in the NPC lines. However, very few biased genes are present in the undifferentiated ESCs. **(b)** Ratio (log2) of the genes that show an allelic bias of 75 % or more towards 129 or Cast in at least one of four undifferentiated 2i ESC RNA-seq profiles (two biological replicas of undifferentiated 2i ES_Tsix-stop and ES_Xist-del, respectively). Similar to the ES_Tsix-stop ESCs, the ES_Xist-del ESCs have a full bias towards one of the two X chromosomes being silenced during XCI (being the Cast-derived X chromosome) due to a deletion in the Xist gene on the 129 allele [64]. **(c)** Localization of the genes as identified in (**b**) over the linear X chromosome.

Suppl Figure 12

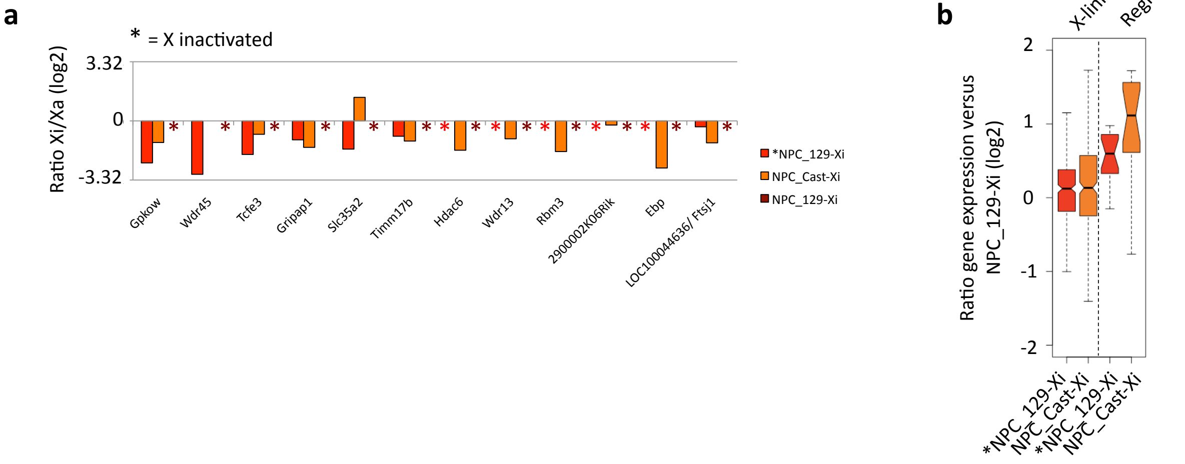

Supplementary Figure 12. Genes within region 1 are significantly higher expressed in the NPCs in which they escape XCI compared with the NPCs in which they are silenced on the Xi.

(a) Xi/Xa Ratio of genes that escape XCI in region 1 in at least one NPC line. A star indicates inactivation of the gene on the Xi. **(b)** Gene expression of *NPC_129-Xi and NPC_Cast-Xi relative to NPC_129-Xi over all X-linked genes (first two boxplots) and over the genes in escape region 1 (last two boxplots; all genes within region 1 of NPC_129-Xi are robustly silenced). The difference in expression level between *NPC_129-Xi and NPC_Cast-Xi in region 1 is caused by the fact that less genes escape XCI in region 1 in *NPC_129-Xi.

Suppl Figure 13

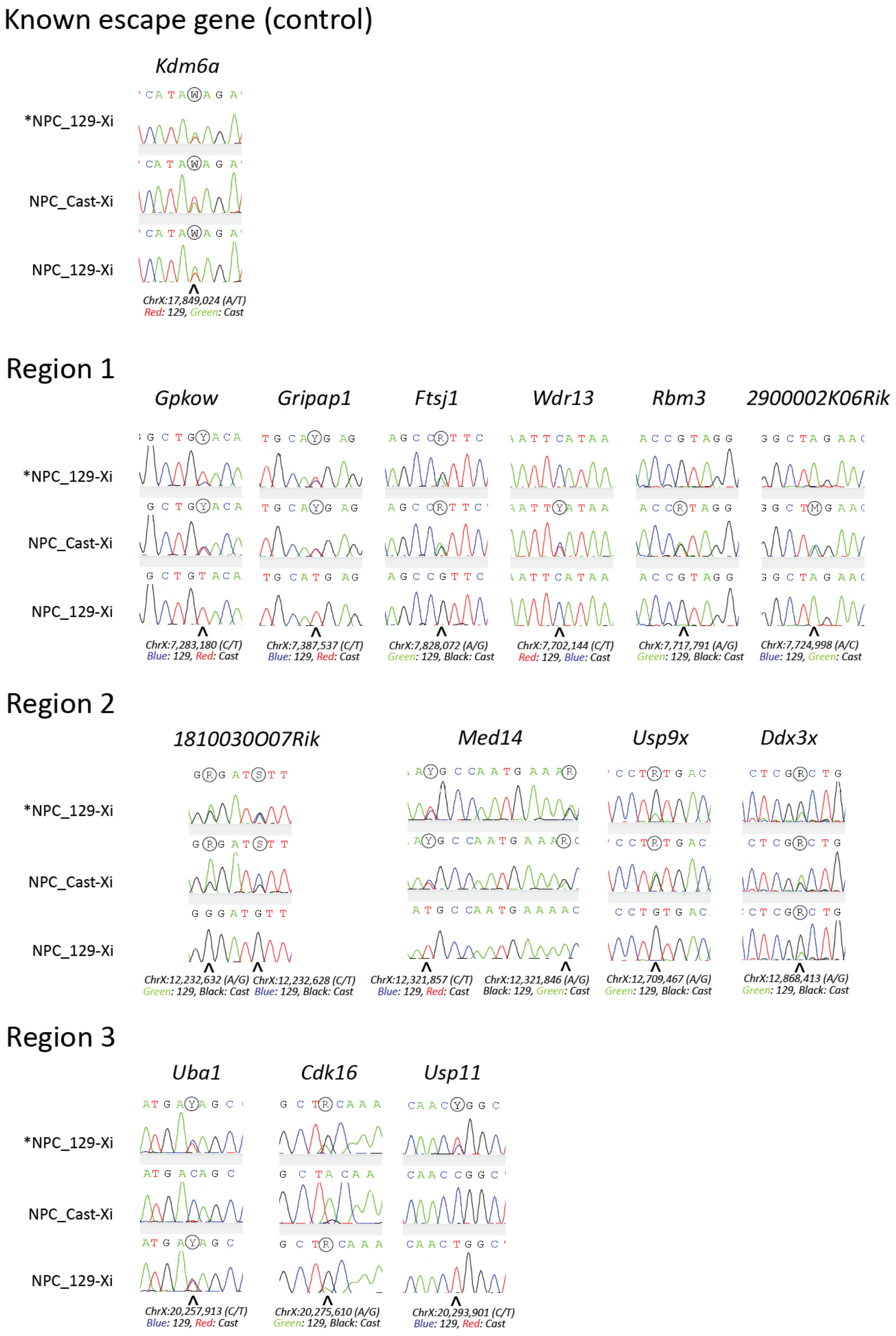

Supplementary Figure 13. Validation of genes escaping XCI in the three escape regions using cDNA Sanger sequencing. In this analysis we included the known escape gene Kdm6a that escapes XCI in all three NPC lines profiled in this study (Table 1), and at least three genes per escape region identified in this study. For each gene, the polymorphic site between 129 and Cast is indicated with an *arrowhead*. Genes that show escape from XCI are indicated with a *circle* around the polymorphic nucleotide. For these nucleotides, there are clear cDNA signals from both 129 and Cast alleles. Except for 1810030O07Rik, the observed patterns of escape using cDNA Sanger sequencing are line with the results obtained by RNA-seq (Table 1).

Suppl Figure 14

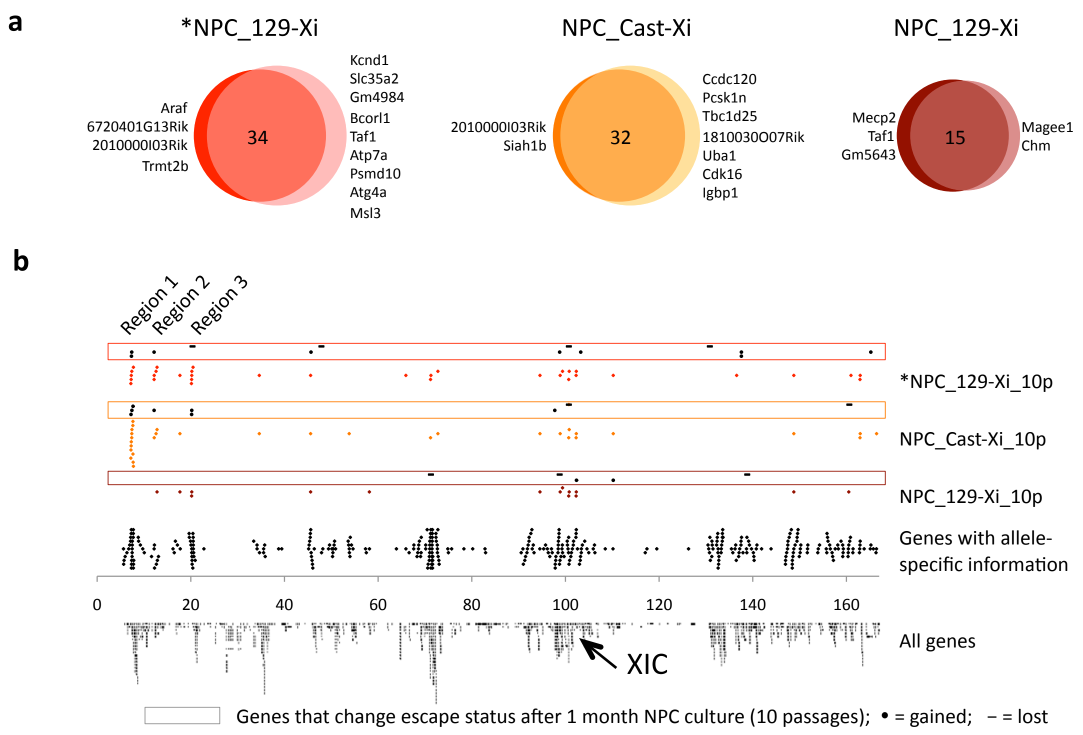

Supplementary Figure 14. The three escape regions are stably maintained during propagation of NPCs. **(a)** Overlap of XCI escape genes before (*left*) and after (*right*) 1 month culturing (ten passages) in each of the NPC lines. **(b)** Localization of escape genes before and after 1 month culturing (10 passages*; 10_p*) over the linear X chromosome. Genes that escape both before as well as after 1 month of culturing are in color, genes that change escape status during culturing are in black within the boxes colored after the respective NPC lines. The *black dots* on the fourth row represent all X-linked genes for which high-confident allele-specific ratios were obtained in NPCs.

Suppl Figure 15

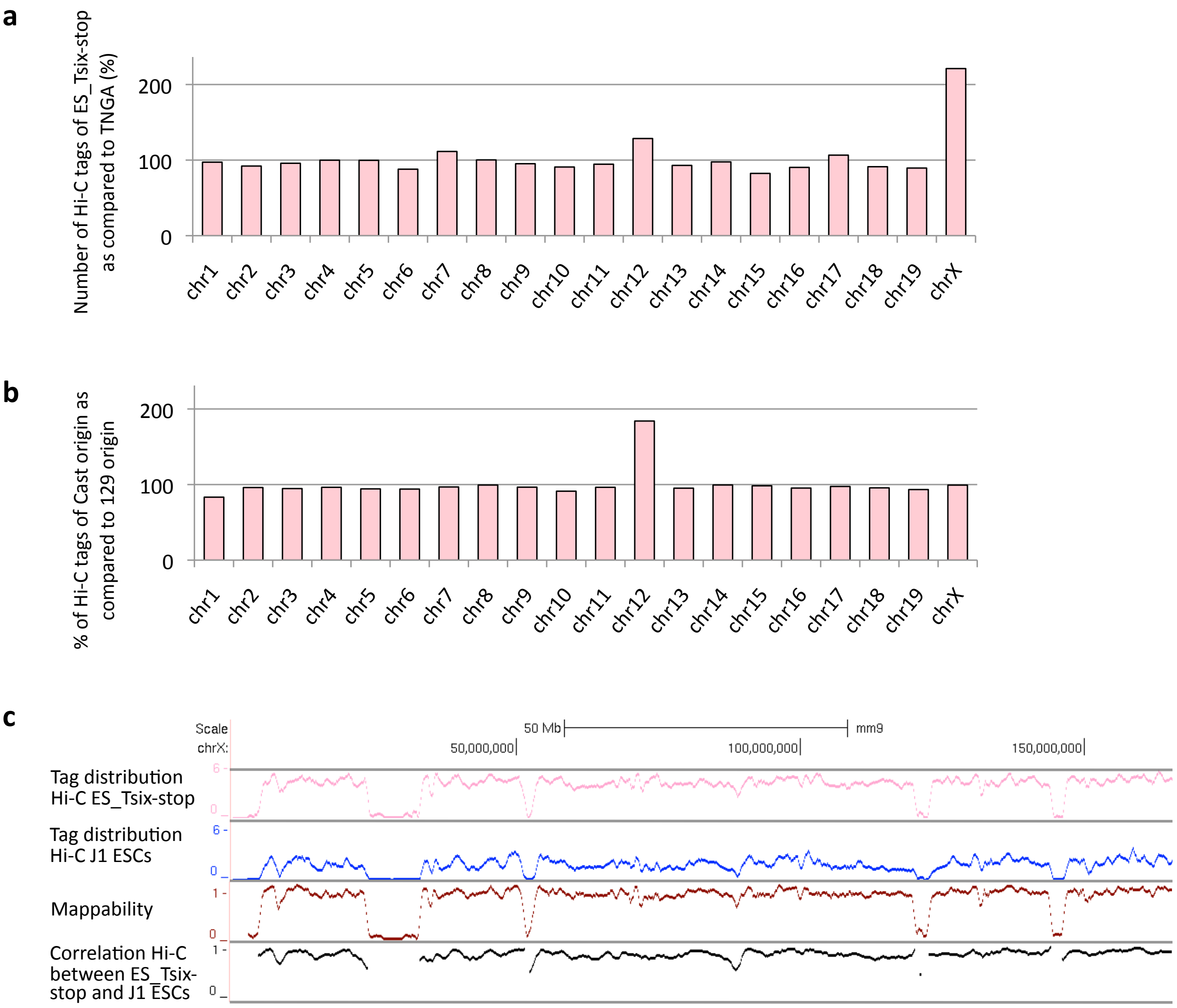

Supplementary Figure 15. Distribution of ES_Tsix-stop Hi-C sequence tags over the genome shows that the ES_Tsix-stop ESCs contains no major genomic abnormalities besides trisomy 12.
(a) Distribution of the Hi-C sequence tags of the female ES_Tsix-stop ESCs over chromosomes compared with male J1 ESCs [51]. As a large number of sequence tags in Hi-C originate from a non-specific background and proximity ligation, this analysis provides an estimate of the karyotype of the ES_Tsix-stop ESCs. As expected, the ES_Tsix-stop ESC line has two times more sequence tags originating from chromosome X compared with the male J1 ESCs. Furthermore, the higher number of tags on chromosome 12 suggests a trisomy 12 present in the ES_Tsix-stop ESC, as also observed in the RNA-seq analysis (Fig. S3a). **(b)** Allele-specific distribution of sequence tags originating from the Hi-C of ES_Tsix-stop ESCs over all chromosomes, plotted as Cast/129 (%). The double number of sequence tags originating from Cast compared with 129 on chromosome 12 shows that the trisomy 12 is caused by an additional chromosome 12 of Cast origin, in line with the RNA-seq analysis (Fig. S3b). The equal distribution of Cast- versus 129-derived tags, together with the tag distribution observed in (**a**) and the RNA-seq tag distribution (Fig. S3), shows that the ES_Tsix-stop ESCs contain no major genomic abnormalities besides trisomy 12. **(c)** Coverage of Hi-C sequence tags of the female ES_Tsix-stop ESCs and male J1 ESCs [51] over chromosome X. Besides non-mappable parts (*third row*) for which no information is present, the ES_Tsix-stop ESCs show a two times higher coverage over the full length of the X chromosome compared with male J1 ESCs, showing that the female ES_Tsix-stop ESCs contains no major chromosomal abnormalities for the X chromosome. Below the Spearman correlations of the 40kb binned Hi-C interaction matrices between female ES_Tsix-stop ESCs and male J1 ESCs [51] over chromosome X (see “Materials and methods” for further details).

Suppl Figure 16

a

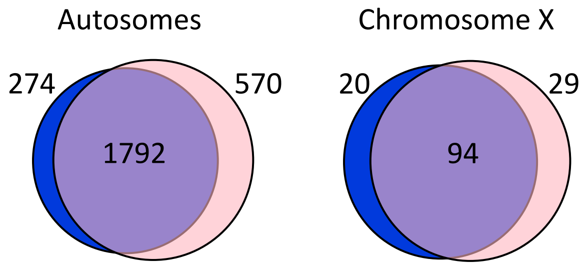

b

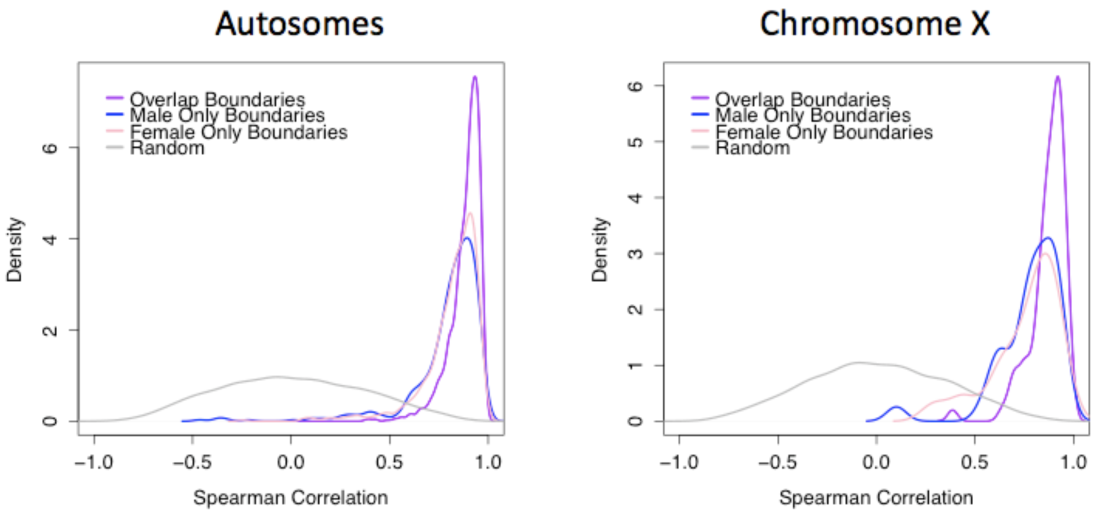

Supplementary Figure 16. Domains and boundaries on autosomes as well as on chromosome X are largely stable between female ES_Tsix-stop and male J1 ESCs [51].

(a) Overlap of topological domain boundaries as determined by Hi-C between male J1 ESCs (*blue*) [51] and female ES_Tsix-stop ESCs (*pink*; this study). The degree of overlap is very similar to the overlap observed between Hi-C replicates [51] **(b)** Density plot of the Spearman correlations of the directionality indices (DIs) between female ES_Tsix-stop and male J1 ESCs [51] at the topological boundary regions. Shared boundaries are in *purple*, in *blue* boundaries only called in male J1 ESCs and in *pink* boundaries only called in female ES_Tsix-stop ESCs. In *grey* are spearman correlations generated for DIs of 40 kb regions randomly sampled from the genome. The Hi-C between the female ES_Tsix-stop and male J1 ESCs is highly correlated at boundary regions, regardless of whether the boundaries are called as shared or as sex-specific.

Suppl Figure 17

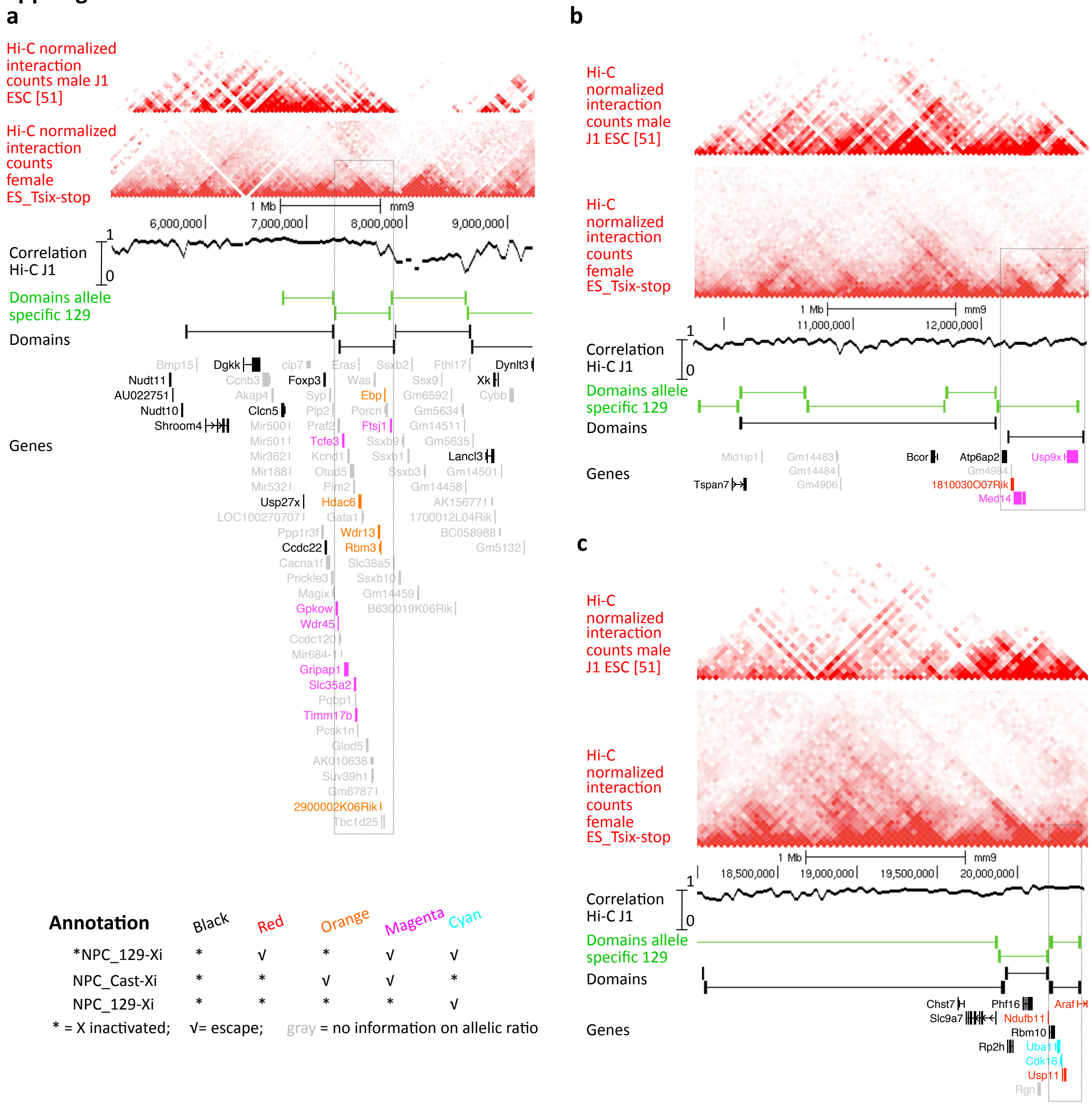

Annotation

	Black	Red	Orange	Magenta	Cyan
*NPC_129-Xi	*	√	*	√	√
NPC_Cast-Xi	*	*	√	√	*
NPC_129-Xi	*	*	*	*	√

* = X inactivated; √= escape; gray = no information on allelic ratio

Supplementary Figure 17. The three regions escaping XCI colocalize with TADs as identified in ES_Tsix-stop ESCs. **(a-c)** Overview of the TADs present at regions 1, 2 and 3 (in (**a**), (**b**) and (**c**), respectively) in the female ES_Tsix-stop ESCs. This figure is the same as Fig. 6, but includes genes for which no information allelic ratios was obtained (mainly due to low expression or the absence of polymorphic sites), as well as the interaction matrix in male J1 ESCs obtained from Dixon et al. [51]. The reason for the absence of data in the male J1 ESCs in the 8-8.5 Mb region in (**a**) is that this is a region of low mappability, in which paired-end 32 nucleotides is not long enough for unique mapping. Since we sequenced paired-end 75 nucleotides, we were able to retrieve information for this region for the ES_Tsix-stop ESCs that we profiled.

Suppl Figure 18

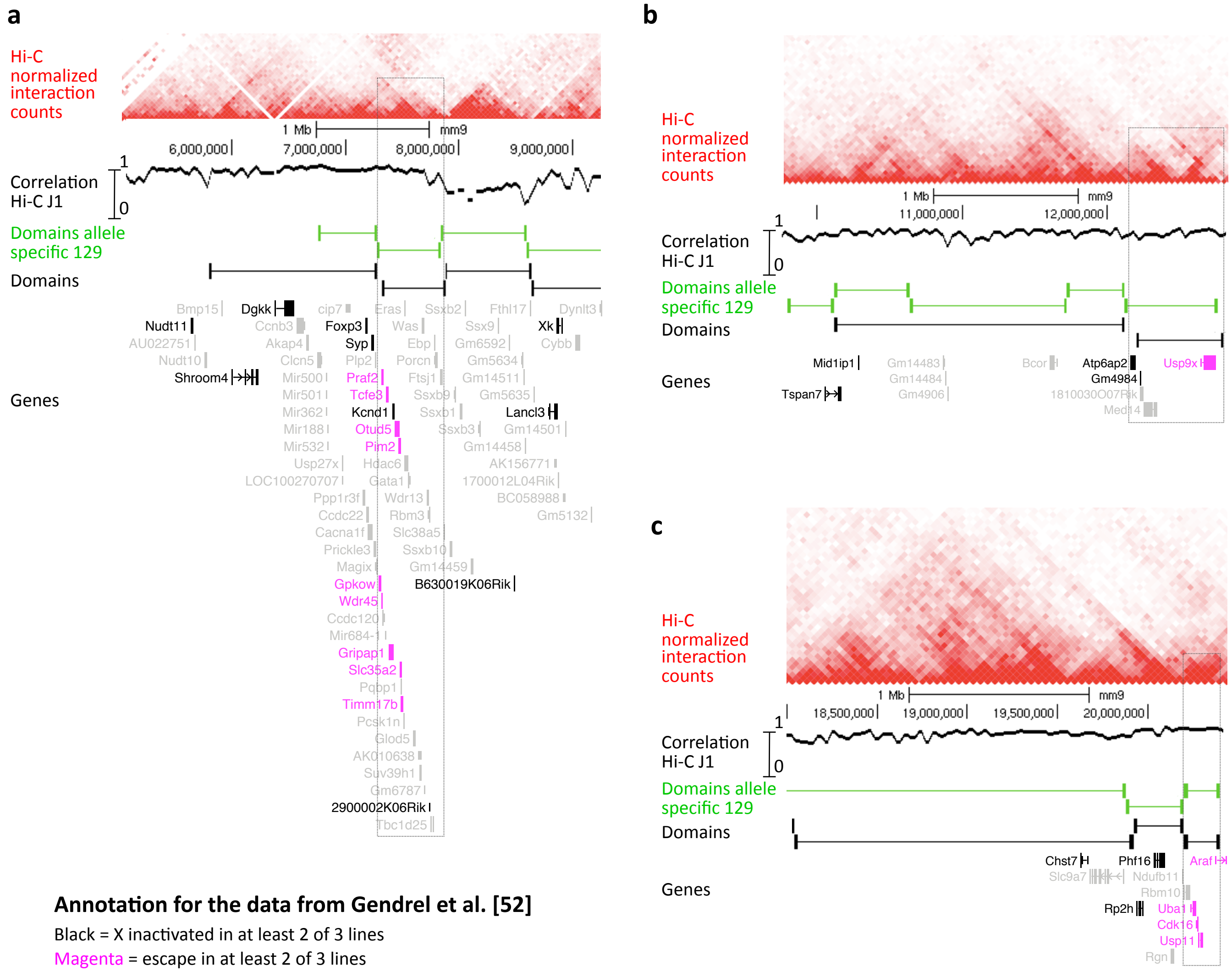

Annotation for the data from Gendrel et al. [52]

Black = X inactivated in at least 2 of 3 lines

Magenta = escape in at least 2 of 3 lines

gray = no information on allelic ratio

Supplementary Figure 18. Validation of the three regions escaping XCI and their association with TADs. **(a-c)** Genes that escape XCI in at least two out of three NPC lines as called using allele specific RNA-seq generated by Gendrel et al. [52] within escape regions 1, 2 and 3 (in (**a**), (**b**) and (**c**), respectively). Escape genes are indicated in *magenta*, genes in *black* are subject to X inactivation. For *gray* genes, no information on allelic ratios was present. The outline of this figure is the same as in Fig. 6 The ES_Tsix-stop ESCs used by Gendrel et al. [52] to generate NPCs are the same as used for the current study. The criteria to call escape genes were similar to those used to call escape genes in the NPCs profiled for the current study.

Suppl Figure 19

a

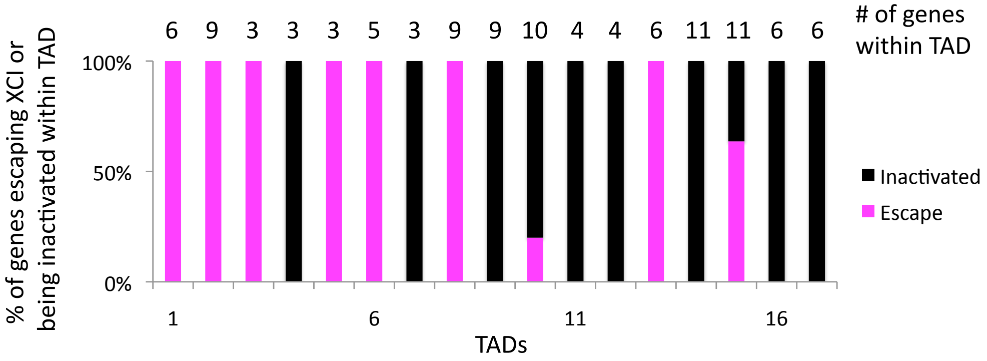

b

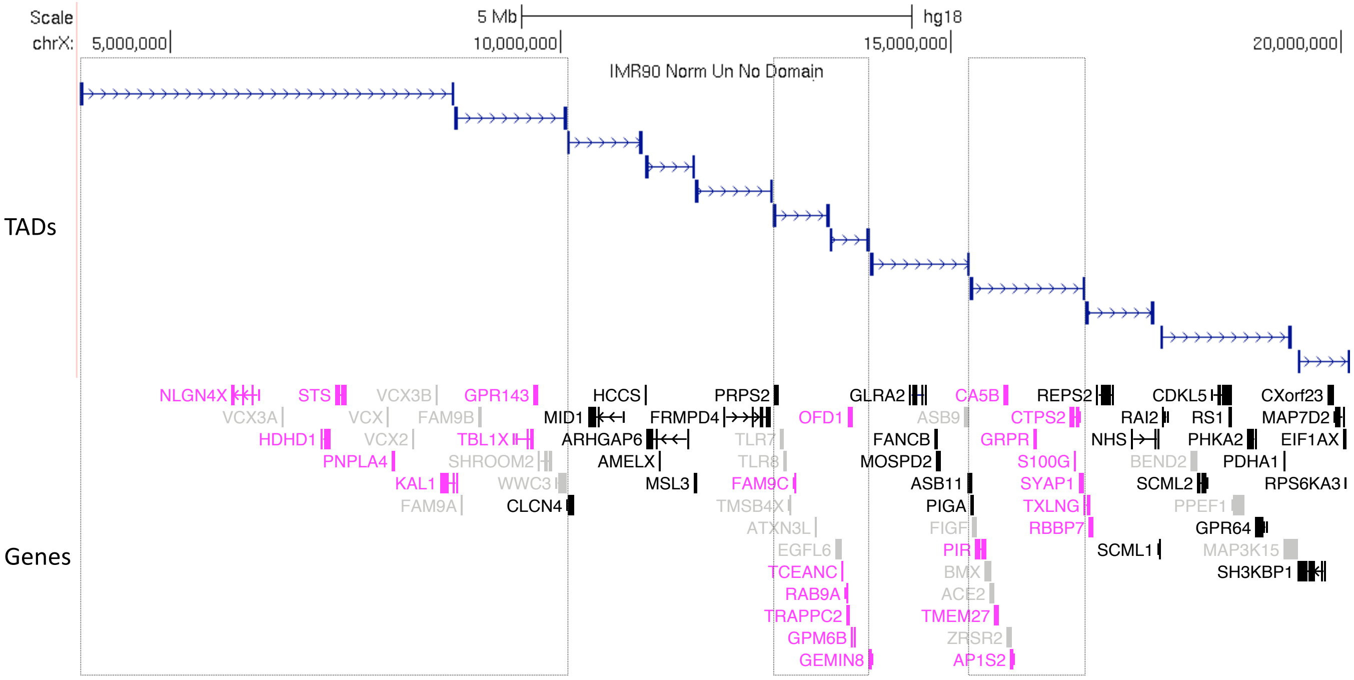

Annotation for the data from Carrel and Willard [29]

Black = X inactivated; Xi expression in 5/9 hybrid clones or less

Magenta = escape; Xi expression in more than 5/9 hybrid clones

gray = no information on allelic ratios

Supplementary Figure 19. In human, clusters of genes escaping XCI on the short arm of chromosome X [29] colocalize with topologically associated domains (TADs) [51].

(a) Percentage of escape (*magenta*) and silenced (*black*) genes within the TADs present on the short arm of chromosome X in human fibroblasts. Only the 17 TADs that contain escape information for more than two of the associated genes are included. The TADs are ranked on the x-axis according to their position on chromosome X, with 1 being very distal from the centromere, and 17 being proximal. For intersection of the genes with TADs, 100kb was subtracted from both sites of all TADs to avoid including genes that have their regulatory sequences in a neighbouring TAD. **(b)** Overview of the chromatin structure (Hi-C) between position 4-20 Mb of chromosome X (including TADs 2 till 9 as present in (**a**)) shows a clear alternating pattern of TADs containing escape genes and TADs containing silenced genes. The legend for genes that escape XCI or genes that are silenced is indicated below.
